# Suppression of CYLD by HER3 confers ovarian cancer platinum resistance via inhibiting apoptosis and by inducing drug efflux

**DOI:** 10.1186/s40164-025-00620-z

**Published:** 2025-02-26

**Authors:** Ye Zhang, Jian-Ge Qiu, Wei Wang, Fan-Li Sun, Xue Wang, Wen-Jing Liu, Xiao-Yu Jia, Hongbin Ji, Lin Wang, Bing-Hua Jiang

**Affiliations:** 1 Department of Gynecology, The Affiliated Cancer Hospital of Zhengzhou University & Henan Cancer Hospital, Zhengzhou University, Zhengzhou, 450000 China; 2 Tianjian Laboratory of Advanced Biomedical Sciences, Academy of Medical Sciences, Zhengzhou University, Zhengzhou, 450000 China; 3 Department of Internal Medicine, The Affiliated Cancer Hospital of Zhengzhou University, Zhengzhou University, Zhengzhou, 450000 China; 4 Shanghai Institute of Biochemistry and Cell Biology, Center for Excellence in Molecular Cell Science, Chinese Academy of Sciences, Shanghai, 200031 China

**Keywords:** Ovarian cancer, CYLD, DDP resistance, HER3, ABCB1

## Abstract

**Background:**

Ovarian cancer (OC) is the most pathogenic gynecological malignant tumor in the world. Due to the difficulty of early diagnosis, most of patients developed chemo-resistance and recurrence during/after chemotherapy.

**Methods:**

CCK8 and flow cytometry were utilized to assess drug sensitivity and apoptosis in parental and drug resistant cell lines. CYLD knockdown or overexpressed cells were employed to investigate its regulatory involvement in DDP resistance. Clinical tumor samples have been utilized to investigate the clinical relevance of CYLD. The drug synergistic effects were investigated through drug combination methods and a nude mice model with ABCB1 inhibitor or HER3 inhibitor.

**Results:**

In this study, we found that CYLD levels were significantly reduced in DDP-resistant cancer tissues and cells compared to the normal tissues and cells. CYLD knockdown in DDP-sensitive cells was sufficient to converse the cells to become DDP resistant by reducing cell apoptosis through increasing Bcl-XL and inhibiting Bax, and by increasing drug efflux via upregulating ABCB1 expression. HER3 expression levels were substantially higher in resistant cancer tissues and cells, and HER3 was the upstream facilitator of suppressing CYLD expression via STAT3 signaling. Furthermore, overexpression of CYLD in resistant cells increased sensitivity to platinum-based chemotherapy both in vitro and in vivo. ABCB1 was a key downstream target of CYLD for regulating tumor growth and therapeutic resistance both in vitro and in vivo, CYLD knockdown promoted the translocation of p65 to nucleus which increased ABCB1 expression through transcriptional activation. High expression levels of HER3 rendered CYLD suppression, consequently, mediated DDP resistance by blocking cell apoptosis pathways and promoting the drug efflux in ovarian cancer.

**Conclusions:**

Our findings identify novel HER3/CYLD/ABCB1 axis that regulate tumor growth and DDP resistance, which may be used as potential novel therapeutic target(s) to overcome ovarian cancer DDP resistance.

**Supplementary Information:**

The online version contains supplementary material available at 10.1186/s40164-025-00620-z.

## Introduction

Ovarian Cancer (OC) is the most lethal gynecological malignancy. Due to the difficulty of early diagnosis [1] and the absence of substantial clinical symptoms [2], 70% patients were detected at advanced stages (III-IV) [3], resulting in ineffective treatment method. The most typical histological type of ovarian cancer is epithelial ovarian carcinoma (EOC) [4], and the standard first-line treatment for EOC is constituted of surgery with the goal of no residual disease (R0) and platinum-based chemotherapy [5]. However, even after careful surgery and sufficient chemotherapy in clinical treatment, the survival percentage for advanced ovarian carcinoma patients still low [6]. The prognosis of patients is closely associated with patients’ response to cisplatin treatment [7], and 19% of early ovarian cancers and 60-85% of recurrent ovarian cancers would eventually develop resistance to platinum [2]. Therefore, to overcome therapy resistance in ovarian cancer is still need studied, with novel molecular processes and therapeutic targets which regulating therapy resistance are urgently need to be elucidated.

Cisplatin (DDP) exerts anticancer activity by generating Pt-DNA adducts and inducing DNA dysfunction [8, 9], which finally leads to cell death and cancer prevention [10]. Preclinical and clinical research revealed that numerous biological processes may be involved in DDP resistance, including enhanced drug efflux, apoptosis suppression, accelerated DNA repair, and modification in drug target genes [11, 12]. The primary mechanisms of therapy resistance in ovarian cancer were suggested as decrease of apoptosis and increase of drug efflux [13]. Recent scientific research has uncovered several other molecular processes in driving platinum resistance, and these findings may help in developing novel treatment strategies. However, new mechanisms of DDP resistance in OC remain to be elucidated, and the most effective combinational therapy strategy for OC needs to be identified.

HER3, a pseudokinase member of the EGFR family encoded by *ERBB3* gene, plays essential functions in tumor growth despite an absence of intrinsic kinase activity [14, 15]. Our earlier investigation suggested that HER3 increased angiogenesis in ovarian cancer progression [16] and was directly regulated by reactive oxygen species (ROS) [17]. Recent research has shown that elevated HER3 expression in breast and lung cancer has a strong association with TKI resistance [18, 19]. In this investigation, we discovered that HER3 was substantially expressed in ovarian cancer tissues, and also involved in DDP resistance process. However, the regulatory role of HER3 in chemotherapeutic resistance of ovarian cancer still unclear, which needs further investigation.

Cylindromatosis gene (CYLD) is one member of lysine 63 deubiquitinases, which can specifically remove the k63 ubiquitin chain. CYLD was firstly discovered as a causative gene of familial cylinaromatosis (an autosomal dominant hereditary disease), and caused CYLD cutaneous syndrome [20–22]. CYLD has been reported as a tumor suppressor to be involved in tumorigenesis and cancer development [20, 23, 24]. Loss of CYLD was associated with poor prognosis through inhibiting cell apoptosis and inducing immune response [25]. In addition, CYLD promoted necroptosis via interacting with RIP1 [26]. However, role of CYLD in OC and platinum resistance is remains unclear.

In this study, we first discovered that CYLD expression was decreased in ovarian cancer tissues. Furthermore, CYLD expression levels were significantly lower in cisplatin-resistant cancer tissues and cells such as A2780-DDP and OVCAR3-DDP cells when compared to normal tissues and parental cells. Herein, we will address several key questions: (1) the role of CYLD in regulating DDP resistance in ovarian cancer; (2) the downstream effector(s) of CYLD that mediate DDP resistance in ovarian cancer; (3) the signal pathways that may be associated with CYLD expression and cancer progression; (4) the upstream regulator of CYLD, and (5) whether there are new small molecular inhibitors or drugs that can be used to reverse DDP resistance in CYLD knockdown cells. Thus, our finding will reveal new mechanism of DDP resistance and identify potential new therapeutic targets to overcome DDP resistance.

## Materials and methods

### Clinical tumor samples and immunohistochemistry

Between January 2017 and December 2020, 33 EOC tumor samples and 10 normal ovarian tissues were obtained from the Biobank of Zhengzhou University’s Affiliated Cancer Hospital (Zhengzhou, China). As previously disclosed, ovarian tumor tissues were collected and maintained in our tissue bank from patients who did not undergo immunotherapy or neoadjuvant chemotherapy prior to surgery [27]. The Biobank also provided normal samples (6 fibroid patients and 4 atypical hyperplasia of endometrial patients). Researchers were unaware of the patient information. Based on the coded information, the EOC patients were staged according to FIGO classification [28], and graded according to World Health Organization standards [29]. Immunohistochemistry (IHC) was carried out as previously described [30], and results were evaluated by the quality of the positive cells and described as IHC scores. Then the scores were categorized into two groups: low expression (scores of 0 to 6), and high expression (scores of 7 to 12) [31].

### Patient samples

#### Case 1

a 57-year-old woman underwent comprehensive staging surgery (hysterectomy, lymphadenectomy, and partial colectomy). The postoperative findings validated the diagnosis of phase IIIc high-grade serous ovarian cancer. From June to November 2018, the patient received six cycles of treatment with cisplatin and docetaxel. According to RECIST v1.1, complete response was obtained, and Ca125 levels were shown to be elevated, but no radiographic picture was altered. This patient’s progress record indicated that he received no treatment throughout this time period. Four months following therapy, the CT imaging revealed a pelvic shadow and higher Ca125 values, indicating platinum resistance recurrence.

#### Case 2

A 46-year-old patient hospitalized for ascites was diagnosed with phase IIIc high-grade serous ovarian cancer following complete staging surgery and received cisplatin and docetaxel treatment in June 2018. After six cycles of chemotherapy, the patient obtained complete response according to RECIST v1.1, and routine reviews were performed every two months, with Ca125 levels remaining unchanged. The CT images revealed the imaging data six months after therapy.There has been no recurrence of OC in these years.

### Cell culture

Human ovarian cancer cell lines Caov3, A2780, A2780/DDP, SKOV3, OVCAR3, OVCAR3/DDP, immortalized ovarian epithelial IOSE386 cell and human embryonic kidney HEK293T cell were cultured in RPMI-1640 or DMEM medium (Gibco, California, USA), supplemented with 10% fetal bovine serum, penicillin and streptomycin. All cells were cultured at 37 °C with 5% CO_2_ incubator.

### RNA isolation and quantitative RT-PCR (qRT-PCR)

Total RNAs from tumor tissues and adherent cells were extracted using TRIzol reagent (Ambion, Texas, USA) according to the manufacturer’s instruction. The quantitative PCR amplifications were performed with ChamQ Universal SYBR qPCR Master Mix (Vazyme, Nanjing, China) using QuantStudio5 system (Applied Biosystems, California, USA). The primer sequences used for quantitative PCR were listed in Supplemental Table 1.

### Western blotting

Total proteins were extracted from tumor tissues or adherent cells, separated by SDS-PAGE and transferred onto PVDF membranes. Then the membranes were blocked with 5% non-fat milk at room temperature and incubated with primary antibodies at 4 °C overnight. After incubation, the membranes were probed by using second antibody, the protein bands were detected with ECL kit (Thermo Fisher scientific, Waltham, USA) [32]. The primary antibodies used in this study were listed in Supplemental Table 3.

### Plasmid construction and lentivirus packaging

The lentiCRISPR v2 plasmid and PLKO-1.0 backbone plasmid were gifts from Professor Zhi Shi of Jinan University (Guangzhou, China). The primer sequences of plasmid constructions used for sgHER3, shCYLD#1 and shCYLD#2 were listed in Supplemental Table 4. The CYLD overexpression plasmid was synthesized by GENE Biotechnology (Shanghai, China), and the HER3-Flag plasmid was synthesized by YouBio Biotechnology (Changsha, China). Lentiviral transfection was conducted following the manufacturer’s instructions. The stable cell lines were selected by puromycin for 2 weeks.

### CCK-8 cell survival assay

Cells were seeded into 96-well plates (5 × 10^3^ per well) and treated with varying dosages of cisplatin, TX1-85-1, or Verapamil as indicated. After 72 h of incubation, OD values were determined using a luminometer at absorbance OD 450 nm with the CCK-8 Kit (Dojindo Laboratories, Shanghai, China), and cell viability levels were calculated based on the OD values.

### Apoptosis assay

Cells were seeded into 6-well plates at a density of 2 × 10^5^/well with various treatments. Cells were collected after 48 h, washed twice with 1x PBS buffer, and stained with an apoptosis detection kit (BD Biosciences, California, USA). Cell fluorescence signals were quantified by flow cytometry (CyFlow Space/Partec, Germany), and the results were analyzed and quantified by using Flow Jo Software.

### Rhodamine 123 accumulation assay

The indicated cells were seeded into the 6-well plates (2 × 10^5^ per well) and treated with different doses of Rhodamine 123 for 2 h at 37 °C. After washed with 1x PBS buffer three times, cells were incubated with 1x PBS at various time periods. The cells were then analyzed by using flow cytometry, and the fluorescence intensity levels were measured by using Flow Jo Software.

### Immunoprecipitation

The cells were seeded into 10 cm plate at a density of 1 × 10^8^cells/plate, and transfected with the HER3-Flag/CYLD plasmids for 48 h. Cells were treated with Immunoprecipitation Kit (Sigma, Missouri, USA) according to the manufacturer’s protocol. The protein samples were separated by 10% SDS PAGE gel, and differential protein bands were detected by enhanced chemiluminescent substrate.

### The synergy of drug combination and quantitative assay

Cells were seeded into 96-well plates (5 × 10^3^ per well) and treated with cisplatin, TX1-85-1, and Verapamil for 72 h, then the cell viability levels were detected. Drug combination effects were evaluated using CompuSyn software (www.combosyn.com), and drug dose-effects were determined using Median Effects techniques. The results were quantified using the Chou-Talalay approach, which included the fractions affected (Fa) and the combination index (CI). The quantitative assay was carried out as previously described [33].

### Chromatin Immunoprecipitation (ChIP) assay

HEK293 cells (1 × 10^7^) were grew in a 6 cm dish and transfected with p65 plasmid for 48 h. The Hyperactive In-Situ ChIP Library Prep Kit for Illumina (Vazyme, Nanjing, China) was used to perform the ChIP test, following the manufacturer’s instructions. Briefly, the cells were harvested and bonded with ConA beads. After the incubation with p65 antibody or IgG, the chromatin was bonded to Hyperactive pA/pG-Tn5 Transposon and extracted. Then the cell’s chromatin samples were detected by qRT-PCR analysis [34].

### Tumor xenograft assay

The 4-week-old female nude mice were obtained from GemPharmatech (Nanjing, Jiangsu, China). The mice were bred in a standard laboratory environment for analysis.

Drug resistance assay: 26 mice were divided into 2 groups randomly. The shNC or shCYLD OVCAR3 cells were resuspended at a density of 5 × 10^6^ cells in serum-free RPMI1640, and injected subcutaneously into the armpit of nude mice. When the tumors were approximately 0.5 cm^3^ in size, the mice were administrated with cisplatin (5 mg/kg, HANSOH PHARMA, Jiangsu, China) in intraperitoneal daily for 5 days, and the tumor sizes were measured every day. The mice were sacrificed at the end of experiments, and tumor tissues were excised and weighted. For the survival assay, the other mice with shNC (*n* = 9) or shCYLD (*n* = 9) xenograft were used to analyze the survival rates of cisplatin treatment until the endpoint of experiment.

Drug resistance reversal assay: Female mice were divided into 3 groups randomly, A2780, A2780-DDP and A2780-DDP CYLD cells were subcutaneously injected into the armpit of mice, respectively, as previously described, then the mice were treated with cisplatin (5 mg/kg, intraperitoneally, daily for 5 days), tumor volumes and tumor weights were analyzed as described above.

Drug combination assay: Female mice were injected by using OVCAR3 cells with CYLD knockdown in the armpit. The mice were randomized into four groups and treated with the following regimens: vehicle alone (0.9% saline), DDP (3 mg/kg, intraperitoneally), Verapamil (25 mg/kg, intraperitoneally), and the combination of DDP and Verapamil daily for 5 days. Then the tumor volumes and tumor weights of each group were analyzed by using the same methods as above.

### Ethics statement

All experiments involving animals were conducted according to the ethical policies and procedures approved by the Animal Ethics Committee of Zhengzhou University (Approval NO.2021060901).

### Statistical analysis

All data were calculated as means ± SD. and analyzed using t-test, and GraphPad Prism 8 software. Survival data were analyzed by the Kaplan-Meier method via SPSS Statistics. The significant differences between overall survival curves were analyzed using log-rank test. P-value at < 0.05 was considered statistically significant.

## Results

### CYLD levels were significantly downregulated in ovarian cancer tissues, and lower CYLD levels were associated with poor prognosis

**Fig. 1:**
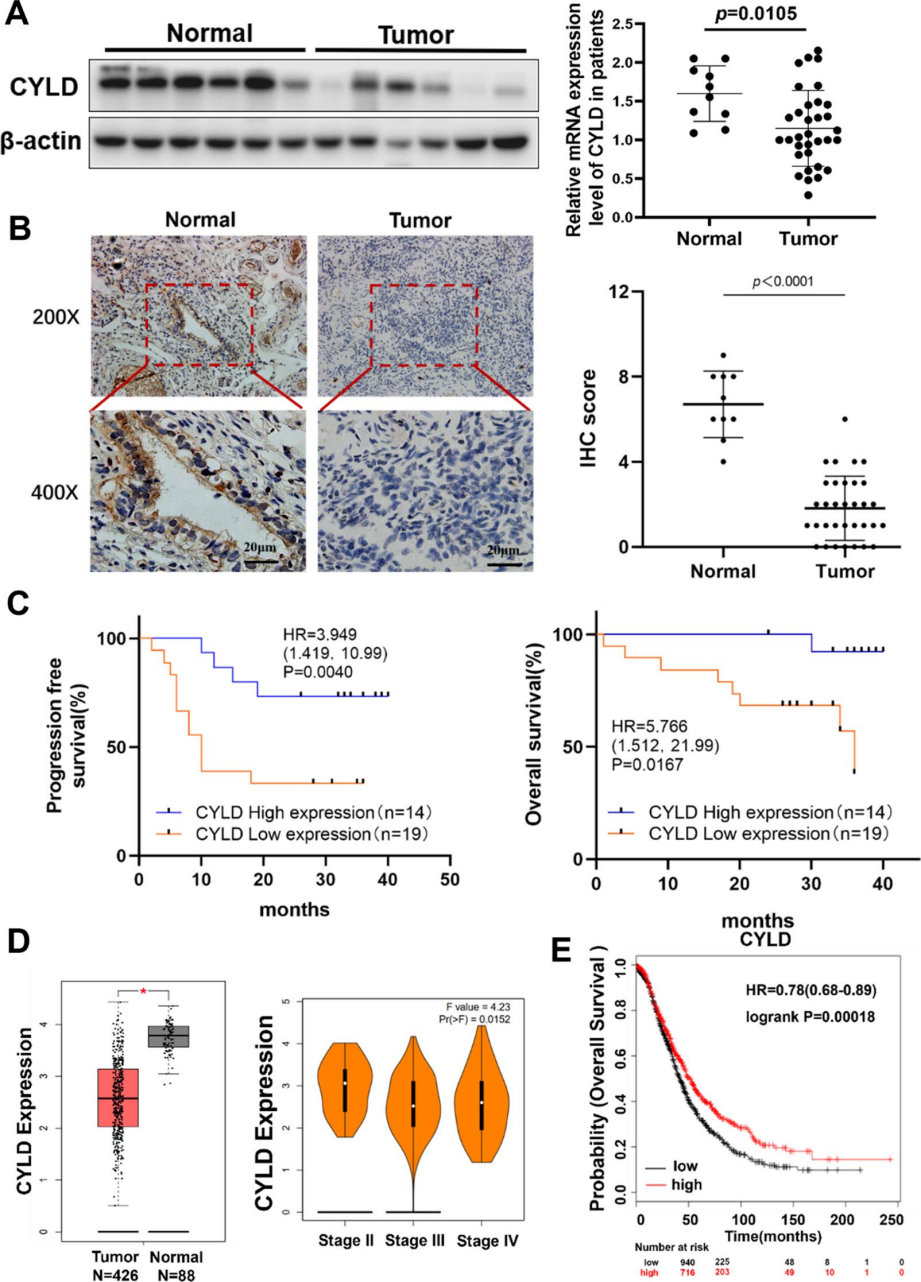
CYLD levels were significantly downregulated in ovarian cancer samples and lower CYLD expression levels were associated with poor prognosis (**A**). CYLD protein and mRNA expression levels were determined using Western blotting and qRT-PCR in normal and tumor tissues gained from the tissue bank. (**B**). IHC staining and scores of CYLD in clinical tumor samples, including normal ovarian tissues (Normal, *n* = 10) and ovarian cancer tissues (Tumor, *n* = 33). Original magnification: 100× or 200×. (**C**). Kaplan-Meier analysis was used to determine the overall survival (OS) and progression-free survival (PFS) of the patient selected from the tissue bank. (**D**). The CYLD expression levels in ovarian cancer patients were evaluated using the GEPIA database (http://gepia.cancer-pku.cn/). The expression levels of CYLD in Grade III were lower than in Grade II. (**E**). The higher levels of CYLD were implied better survival rate in ovarian cancer patients as suggested in Kaplan-Meier Plotter database (http://kmplot.com/analysis/index.php?p=background). Data were statistically analyzed with Student’s t-test and values are shown as mean ± SD. *indicates significant difference at *p* < 0.05

To evaluate the significance of CYLD expression in carcinogenesis, the expression levels of CYLD were discovered in several types of malignancies using the UCSC Xena database, and the results demonstrated that CYLD levels were greatly downregulated in the majority of cancers, particularly ovarian cancer (Fig. S1A). Then, CYLD expression levels were evaluated in normal ovarian and epithelial ovarian cancer tissues, and both CYLD protein and mRNA expression levels were substantially reduced in tumor tissues compared to normal tissues (Fig. 1A). In addition, we utilized immunohistochemistry (IHC) staining, and found the CYLD expression levels and IHC sores in tumor tissues were much lower than those in normal tissues (Fig. 1B). We then studied the relationship between CYLD expression levels and clinicopathological characteristics of ovarian tumor tissues and found that there was no significant correlation of CYLD expression levels with clinical factors such as age, ascites volume, and metastatic site. Interestingly we found that CYLD expression levels were decreased in clinical most advanced stages of cancer, and higher levels of CYLD predicted a better response to cisplatin treatment (Supplementary Table 1). Patients with lower CYLD expression levels had much worse outcomes than those with higher CYLD expression levels (Fig. 1C). Furthermore, we analyzed CYLD expression levels in the GEPIA database, and the results revealed that CYLD was a tumor suppressor in ovarian cancer, and CYLD expression levels were reduced in clinical advanced stages of cancer patients (Fig. 1D). The predictive roles of CYLD levels in ovarian cancer were further investigated using the Kaplan-Meier plotter database, the results showed that patients with higher CYLD expression levels had a better overall survival rate (Fig. 1E), and the correlation of different CYLD expression levels with progression free survival of patients was similar to the correlation with the overall survival (data not shown). We further analyzed the protein expression levels of CYLD in the ovarian cancer tissues through CPTAC database, and the result showed that CYLD protein levels were similarly reduced in a different cohort of ovarian cancer tissues (Fig. S1B). Thus, this result demonstrated that CYLD was a tumor suppressor in ovarian cancer, and patients with lower CYLD levels had poor prognosis.

### CYLD was a new biomarker and DDP sensitizer

**Fig. 2:**
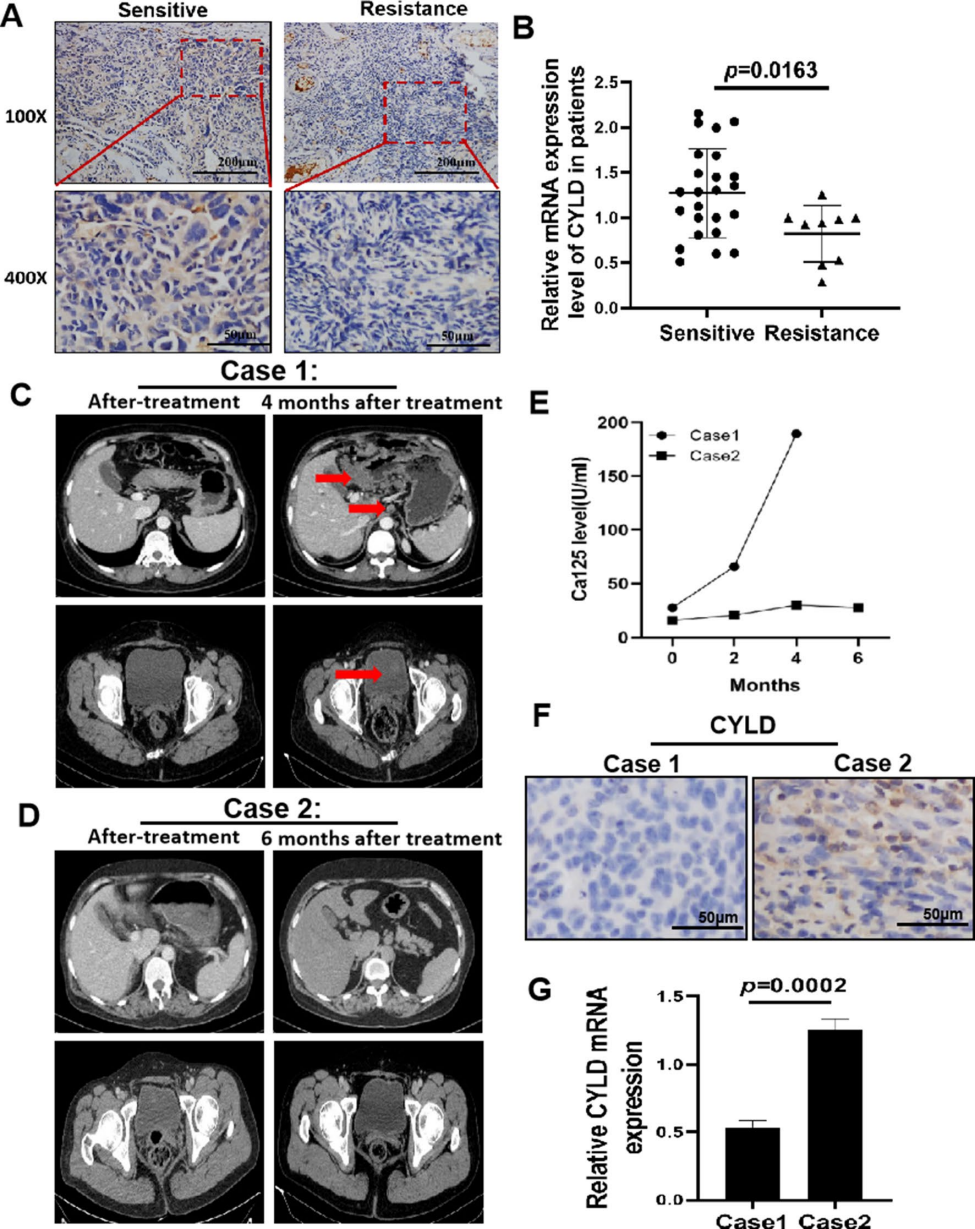
CYLD is an essential DDP sensitizer and potential new biomarker (**A**). IHC staining of CYLD in tumor samples at original magnification of 100× or 400×, including cisplatin sensitive (*n* = 24) and resistant (*n* = 9) tissues. (**B**). The mRNA expression levels of CYLD in sensitive/resistant groups were determined using qRT-PCR and displayed in a scatter plot. (**C**,** D**) CT images of Case 1 and Case 2 patients from Biobank at the end of treatment and four to six months later. The red arrow indicates relapsed malignancies. (**E**). The Ca125 levels of Case 1 and Case 2 patients were measured at each review time; the 0 represented the time when the previous treatment was completed. (**F**). CYLD staining signals in Case 1 and Case 2 tumor specimens. (**G**). The mRNA expression levels of CYLD in tumor samples from Case 1 and Case 2 patients were examined. Magnification: 200×

Because the resistance to DDP treatment is a major clinical issue in ovarian cancer, we wondered that CYLD expression could be linked to cisplatin resistance, and found the expression levels of CYLD were much lower in cancer tissues of the cisplatin resistant group than in the sensitive group (Fig. 2A). CYLD mRNA expression levels were also much lower in resistant individuals than sensitive ones (Fig. 2B). We specifically used two cases (Case 1 from the cisplatin resistant group and Case 2 from the cisplatin sensitive group) to highlight the importance of CYLD expression in DDP resistance in ovarian cancer. We exhibited computed tomography (CT) scans of two clinical patients: Case 1 displayed a pelvic shadow four months after DDP therapy (Fig. 2C, right panel), and Case 2 demonstrated no recurrence six months later (Fig. 2D, right panel). The serum biomarker Ca125 levels along with an imaging scan were subsequently monitored every two months. Two months later, Ca125 levels were higher in Case 1, but not altered in Case 2, indicating that platinum resistance had reoccurred in Case 1 (Fig. 2E). Furthermore, CYLD expression was lower in Case 1 with platinum resistance recurrence, but increased in the platinum-sensitive Case 2 patient (Fig. 2G). CYLD mRNA expression levels were greatly lower in patients with platinum-resistant cases (Fig. 2F). These results demonstrated that CYLD expression levels were decreased in ovarian cancer patients as they gained resistance to DDP treatment, suggesting that CYLD is a DDP sensitizer and new biomarker of ovarian cancer DDP resistance.

### CYLD knockdown rendered cells resistant to DDP treatment, while forced expression of CYLD in DDP resistant cells enhanced cell sensitivity to DDP treatment

**Fig. 3:**
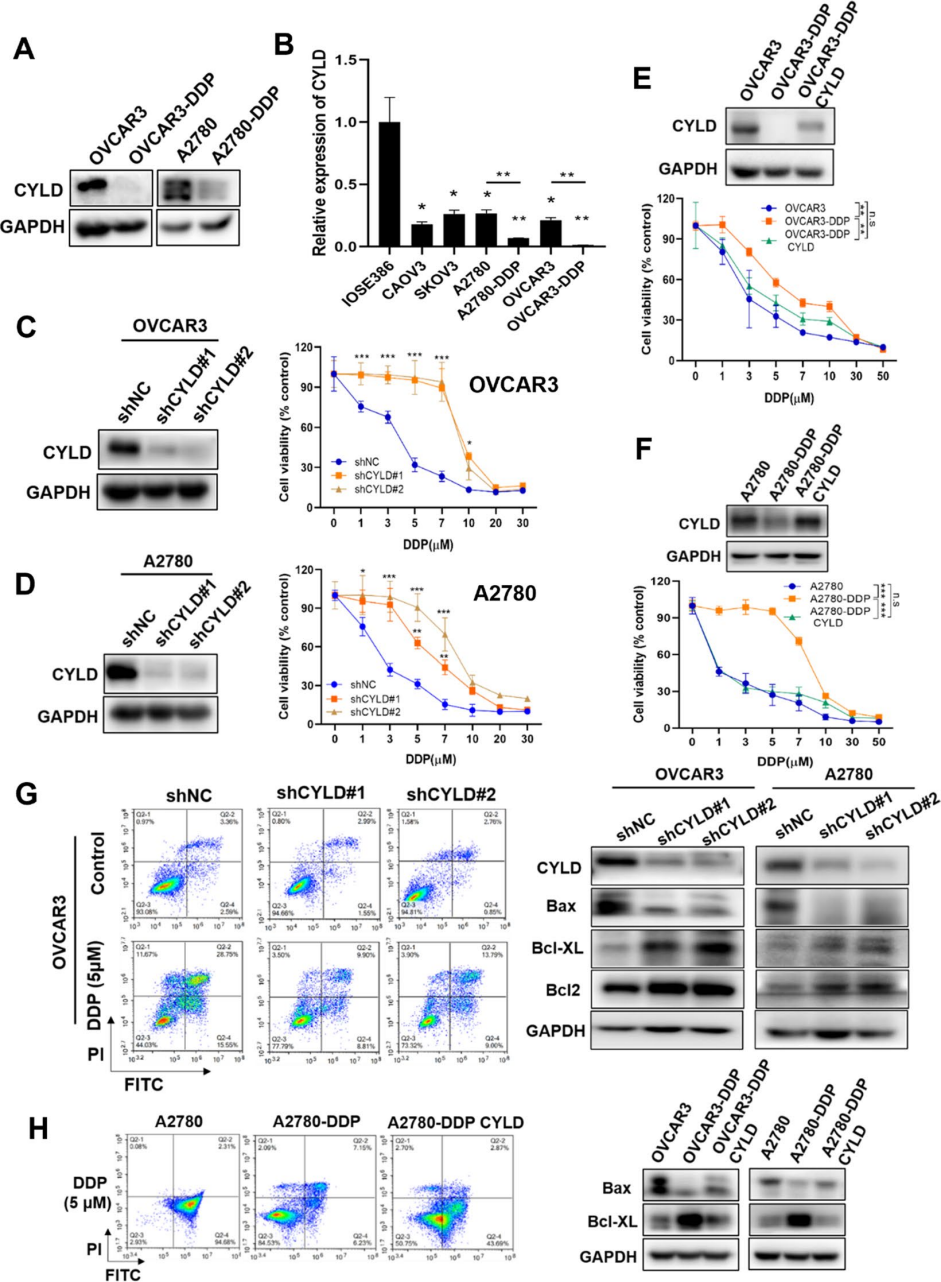
CYLD knockdown made DDP sensitive cells to become resistant, whereas forced expression of CYLD in DDP resistant cell lines made the cells to become DDP sensitive (**A**). Western blotting was used to detect the expression levels of CYLD in OVCAR3/OVCAR3-DDP and A2780/A2780-DDP. (**B**). The mRNA expression levels of CYLD in a human immortalized ovarian cell line (IOSE386), four human ovarian cancer cell lines (Caov3, SKOV3, A2780, and OVCAR3), and two DDP resistance ovarian cancer cell lines (A2780-DDP and OVCAR3-DDP) were identified using qRT-PCR. (**C**,** D**). Western blotting was used to quantify CYLD expression levels in stable CYLD knockdown OVCAR3 or A2780 cell lines, and the DDP sensitivity levels were determined using the CCK8 assay in CYLD knockdown cells. (**E**,** F**). The protein expression levels of CYLD in the identified cell lines were examined using Western blotting, and the DDP sensitivities in these three cell lines were assessed using the CCK8 kit. (**G**,** H**). After 48 h of incubation with 5µM DDP or PBS, cell apoptosis rates in shCYLD cells were compared to control cells or A2780/A2780-DDP/A2780-DDP with CYLD overexpressed cell lines. The total percentage of early and late apoptosis rates was shown. The apoptosis-related proteins were identified using Western blotting. Data were statistically analyzed with Student’s t-test and values are shown as mean ± SD. *, **, *** indicates significant difference at *p* < 0.05, *p* < 0.01, *p* < 0.001, respectively

To determine the expression levels of CYLD in DDP resistance in ovarian cancer, we investigated parental and DDP resistant cell lines (OVCAR3/OVCAR3-DDP, A2780/A2780-DDP) and discovered that CYLD protein expression levels were dramatically reduced in DDP resistant cells (Fig. 3A). Meanwhile, several OC cell lines (Caov3, SKOV3, A2780, and OVCAR3) have lower CYLD levels than the normal IOSE386 cell line (Fig. 3B). These findings revealed that CYLD expression levels were reduced during the progression of DDP resistance. But it remains to be confirmed whether lower CYLD expression could be an early component of DDP resistance. To evaluate if CYLD suppression is sufficient to alter chemoresistance, we knocked down CYLD expression in OVCAR3 and A2780 cells. We discovered that inhibition of CYLD was sufficient to promote cell resistance to DDP treatment (Fig. 3C and 3D). In addition, we generated CYLD-overexpressed OVCAR3-DDP and A2780-DDP cells, and demonstrated that forced expression of CYLD was sufficient to increase cell sensitivity to DDP treatment, converting the DDP resistant cells to sensitive cells (Fig. 3E and 3F). These results revealed that CYLD was critical in controlling DDP resistance in ovarian cancer cells.

Then we evaluated whether CYLD affected DDP resistance by modulating cellular apoptosis. We applied GSEA analysis on ovarian cancer specimens obtained from the GEO dataset (GSE9891), and showed that higher CYLD expression levels were associated with activation of apoptotic signaling (Fig. S2A). Cell apoptosis rates were examined in OVCAR3 and A2780 cells treated with 5µM DDP or PBS buffer. The percentages of apoptotic cells were greatly reduced in the CYLD knockdown group treated with DDP than those in the control (Fig. 3G and Figs. S2B-S2C). Furthermore, we discovered that the protein expression levels of the pro-apoptosis factor Bax were reduced in the CYLD knockdown group, whereas the anti-apoptotic proteins Bcl-XL and Bcl-2 were greatly increased (Fig. 3G). Furthermore, the proportions of apoptotic cells in A2780-DDP with CYLD overexpression group were significantly higher than those of A2780-DDP, and the similar results were obtained in A2780 cells with or without CYLD overexpression (Fig. 3H and Fig. S2D). CYLD did not influence the expression levels of RIP3 (necrosis marker), p62 and ATG5 (autophagy marker), as well as levels of GPX4 and SLC7A11 (ferroptosis marker) in ovarian cancer A2780 and OVCAR3 cell lines (Fig. S2E). Further studies suggested that compared to OVCAR3-DDP/A2780-DDP, levels of Bax were upregulated in CYLD-overexpressed resistant cells and OVCAR3/A2780 cells, whereas levels of Bcl-XL were downregulated in the cells, respectively (Fig. 3H). These results showed that CYLD mediated DDP resistance through regulating apoptosis via Bax/Bcl-XL signals.

### CYLD knockdown promoted drug efflux through regulating ABCB1 expression

**Fig. 4:**
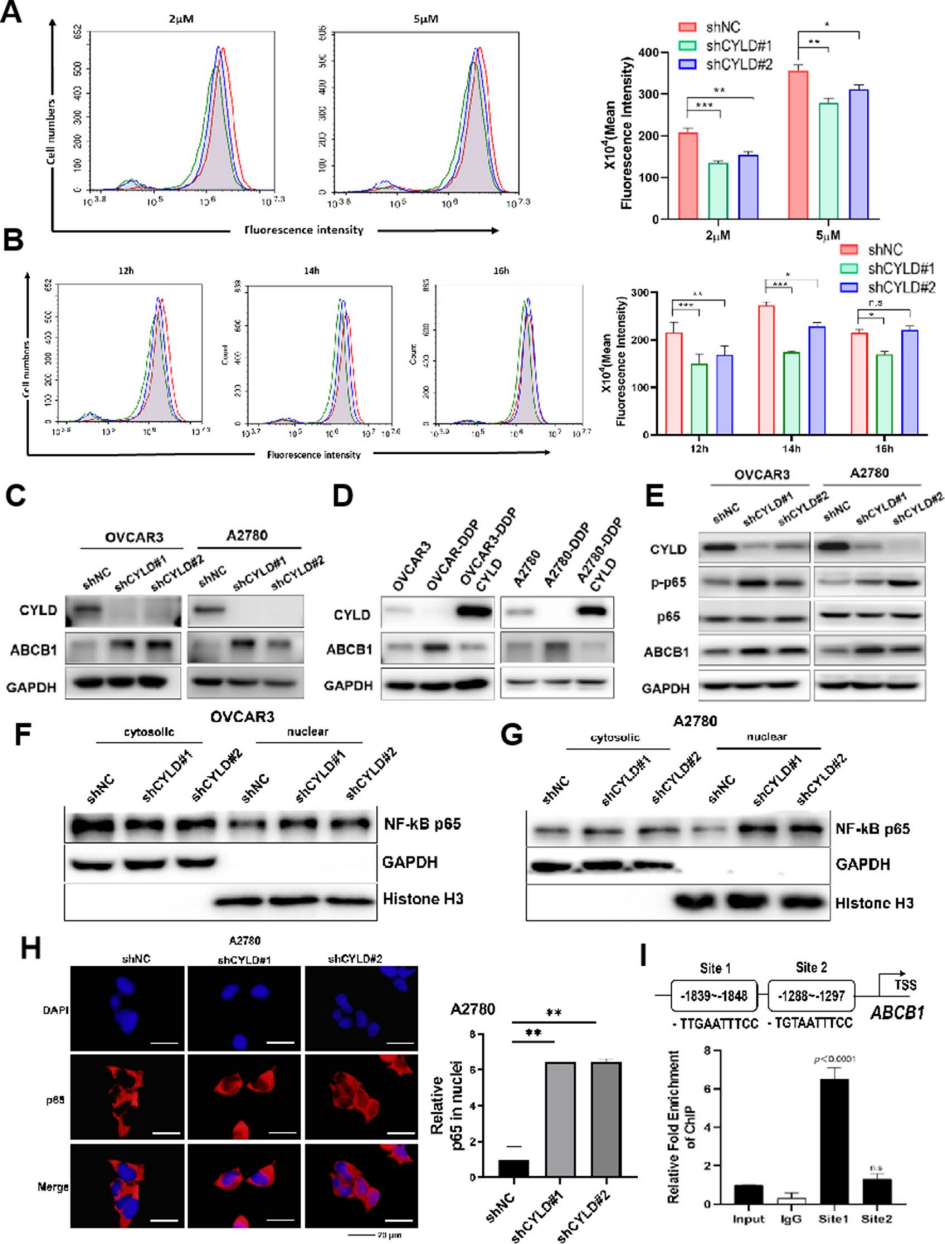
CYLD knockdown promoted drug efflux through NF-κB p65 to induce ABCB1 expression (**A**) The shCYLD or shNC OVCAR3 cells were incubated with PBS for 12 h at 37 °C following pre-treatment with 2µM or 5µM Rhodamine 123 for 2 h. Rhodamine 123 accumulation in cells was evaluated using flow cytometry. The quantitative data were presented. (**B**) The shCYLD or shNC OVCAR3 cells were incubated with PBS for different time points (12 h, 14 h, and 16 h) at 37 °C after being pre-treated with 2µM Rhodamine 123 for 2 h. The accumulation of Rhodamine 123 in cells was determined by Flow Cytometry. (**C**,** D**). Western blotting was used to detect the levels of ABCB1 protein expression in CYLD knockdown and overexpressed OVCAR3/A2780-DDP cell lines. (**E**). Western blotting was used to examine the protein levels of p-p65 in CYLD silenced OVCAR3 and A2780 cells. (**F**,** G**). In OVCAR3 and A2780 cells, CYLD knockdown led to NF-κB p65 subunit translocation to the nucleus. (**H**). CYLD shRNAs increased the nuclear position of NF-κB p65 as observed through immunofluorescence. We randomly counted the total cell number and cell nucleus number with p65 staining signal in each photo, then p65 staining cell nucleus number divided by total cell number which was further normalized to those of the control to obtain the relative nuclear entry of p65. (**I**). The possible binding sites and p65 motif in the ABCB1 promoter region were identified, and ChIP tests were used to investigate binding. Data were statistically analyzed with Student’s t-test and values are shown as mean ± SD. *, **, *** indicates significant difference at *p* < 0.05, *p* < 0.01, *p* < 0.001, respectively

In light of the fact that the effect of CYLD-regulated cell death in DDP-sensitive cells may be regulated by other mechanism in the cells [21, 35], we explored other alternative molecular regulatory pathways that may contribute to drug resistance in ovarian cancer. To assess transporter activity in parental OVCAR3 and CYLD knockdown cells, we employed internal levels of Rhodamine 123, a fluorescent dye that ABC transporters can carry across the cell membrane. Incubating CYLD knockdown cells with different concentrations of Rhodamine 123 resulted in dramatically increased intracellular fluorescence intensity compared to the control group, especially at 2 µM (Fig. 4A). We then examined the intracellular fluorescence intensities at various time points after the Rhodamine 123 incubation with the cells. The results showed that intracellular fluorescence values were dramatically reduced in the CYLD knockdown cells after 12 h, but not after 16 h (Fig. 4B). These data suggested that CYLD inhibition increased the influx of Rhodamine 123 into cells. To get insight into the potential targets of CYLD in the ABC transporter superfamily, we assessed the expression levels of several standard ABC transporters in CYLD knockdown cells. We discovered that the expression levels of ABCB1 were dramatically increased in CYLD-knockdown cells (Fig. 4C), whereas the expression levels of ABCC1, ABCG2, and ABCG9 did not significantly change (Figs. S3A-S3B). Furthermore, ABCB1 expression levels were significantly higher in OVCAR3-DDP/A2780-DDP cells than those in CYLD-overexpressed resistant cells and OVCAR3/A2780 cells (Fig. 4D). CYLD may be a deubiquitinase, we further investigated whether it regulated ABCB1 expression by protein ubiquitination, and found there was no direct interaction between CYLD and ABCB1, implying that CYLD may indirectly influence ABCB1 expression levels (Fig. S3C). We have analyzed the relationship between CYLD and ABCB1 levels in ovarian cancer samples, and found a significant negative correlation between the expression levels of ABCB1 and levels of CYLD (Fig. S3D). Previous research linked CYLD loss to drug resistance via NF-κB hyperactivation [23, 24, 36, 37]. We discovered that CYLD knockdown cells had considerably higher levels of p-65 (ser536), a crucial intermediate signaling molecule (Fig. 4E).

To find out if CYLD knockdown boosted NF-κB p65 translocation to the nucleus, we extracted cytoplasm and nucleus proteins, and nuclear extracts from CYLD knockdown cells indicated much higher levels of p65 than the control (Fig. 4F and 4G). Then, we utilized immunofluorescence to evaluate p65 levels within the cells, and found that CYLD knockdown increased p65 signals inside the nucleus, indicating the p65 subunit’s translocation to the nucleus (Fig. 4H and Fig. S3E). We also investigated the transcriptional activities of NF-κB using a luciferase reporter. The NF-κB luciferase reporter assay was used to evaluate p65 transcriptional activities in control and CYLD knockdown cells, and normalized to Dual-Luciferase reporter activity levels in the cells. We showed that the NF-κB luciferase activities were significantly increased in CYLD knockdown cells compared to the control (Figs. S3F-S3G). We further showed two potential binding sites of p65 in the promoter region of ABCB1 as predicted through JASPAR program, the ChIP assay and luciferase reporter assay showed that the direct binding site of p65 in the ABCB1 promoter region was at the Site 1 (~ 1839–1848) (Fig. 4I and Figs. S4A-S4B). Thus, ABCB1 was a direct downstream target of CYLD for mediating DDP resistance.

### HER3 negatively regulated CYLD expression via phosphorylation of STAT3

**Fig. 5:**
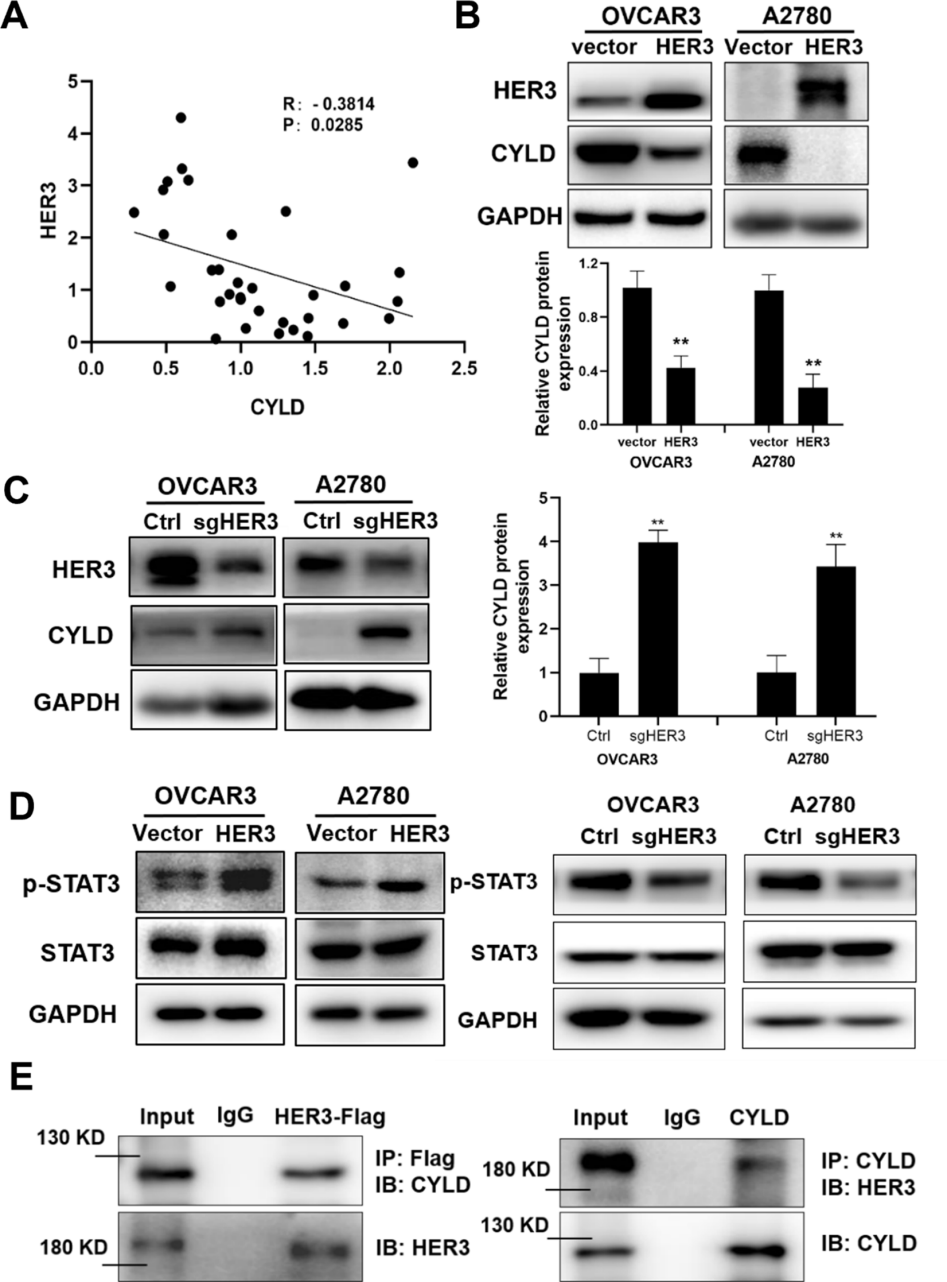
HER3 inhibited CYLD expression via phosphorylation of STAT3 (**A**). Spearman correlation analysis was used to examine the relationship between CYLD and HER3 levels in ovarian cancer samples. (**B**,** C**). Western blotting was used to detect CYLD expression levels in HER3-silenced or overexpressed OVCAR3 and A2780 cells. (**D**). Western blotting was used to identify p-STAT3 expression levels in HER3-silenced or overexpressed OVCAR3 and A2780 cells. (**E**). Following HER3 and CYLD overexpression in HEK293T cells, HER3 and CYLD proteins were immunoprecipitated with HER3 or CYLD antibodies, which were then identified and evaluated by Western blotting. Data were statistically analyzed with Student’s t-test and values are shown as mean ± SD. *, **, *** indicates significant difference at *p* < 0.05, *p* < 0.01, *p* < 0.001, respectively

Our previous studies showed that the expression levels of HER3 were increased in ovarian tumors and were associated with the drug resistance. In addition, we found that ROS induced higher expression levels of HER3 in ovarian cancer and played an important role in tumor development and angiogenesis [38]. Recent studies found that HER3 activated several pathways such as PI3K/AKT, MEK/MAPK, Jak/STAT, and Src kinase, promoting the occurrence and development of tumors, which may play a crucial role in the resistance to various TKi drugs [39]. Thus, to investigate the potential mechanism of low CYLD levels in ovarian cancer DDP resistance, we selected to test roles of HER family expression in the study. We initially measured the mRNA expression levels of the HER family in ovarian tumor tissues, which include EGFR, HER2, HER3, and HER4, and found a significant negative correlation between the expression levels of HER3 and levels of CYLD (Fig. 5A), but not levels of other HER family members (Fig. S5A). We hypothesized that HER3 may be an upstream modulator of CYLD in ovarian cancer. To determine the role of HER3 in CYLD regulation, we evaluated CYLD expression levels in HER3-overexpressing ovarian cancer cells and showed that CYLD expression levels were much reduced in HER3-overexpressed OVCAR3/A2780 cells (Fig. 5B). In addition, the expression levels of CYLD were increased in OVCAR3/A2780 cells with HER3 knockdown compared to control cells (Fig. 5C). These findings further revealed that HER3 negatively affected CYLD expression levels. For further insight into how HER3 reduced CYLD expression, we assessed the activation levels of many important regulatory proteins in HER3-overexpressed cells, and showed that there were considerably higher levels of crucial intermediate signaling molecule p-STAT3 (T705) when compared to control cells (Fig. 5D), whereas other possible molecules such as p-c-Jun, p-p38, and p-Erk1/2 were not significantly changed (Fig. S5B). Furthermore, p-STAT3 expression levels in HER3-silenced OVCAR3/A2780 cells were significantly reduced in the cells (Fig. 5D). STAT3 knockdown or treatment of STAT3 inhibitor STAT3-IN-1 in A2780 and OVCAR3 cells induced CYLD expression (Fig. S5C). Additionally, the immunoprecipitation assay showed that HER3 may directly bind and interact with CYLD (Fig. 5E). Thus, our findings showed that HER3 decreased CYLD expression levels via activating STAT3 signaling.

### HER3 functioned as a crucial upstream regulator of CYLD in DDP resistance

**Fig. 6:**
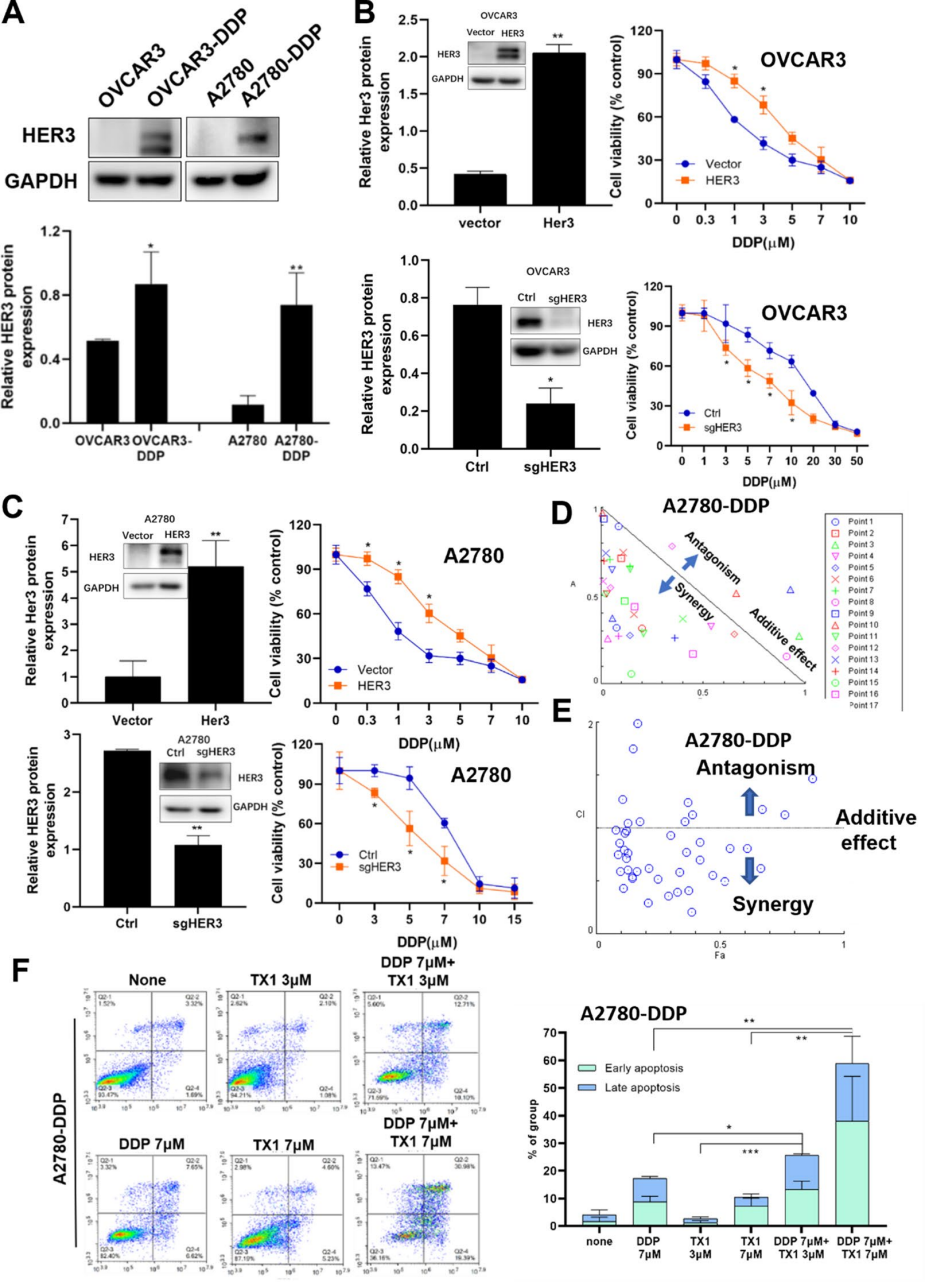
HER3 was important upstream regulator of CYLD for mediating DDP resistance and HER3 inhibitor made the DDP resistant cells to become sensitivity again (**A**). HER3 protein expression levels in OVCAR3-DDP/A2780-DDP cells were determined by Western blotting. (**B**,** C**). The CCK8 assay was used to assess the DDP sensitivities in HER3-silenced/HER3-overexpressed OVCAR3 and A2780 cells. (**D**,** E**). The drug combination analysis of DDP and TX1-85-1 in A2780-DDP cells was performed using the CompuSyn program. The dose-effect curve and Fa-CI plots were presented. DDP (7µM) and TX1-85-1 (3µM and 7µM) yielded CI values of 0.7494 and 0.8557. (**F**). Flow cytometry was used to identify cell apoptosis in A2780-DDP cells treated with DDP (7µM) and TX1-85-1 (3µM and 7µM). The apoptosis rates were represented by the total percentage of early and late apoptosis. Data were statistically analyzed with Student’s t-test and values are shown as mean ± SD. *, **, *** indicates significant difference at *p* < 0.05, *p* < 0.01, *p* < 0.001, respectively

In order to explore the role of HER3 in DDP resistance in ovarian cancer, we assessed HER3 expression levels in OVCAR-DDP and A2780-DDP cells, and found that HER3 levels were significantly higher than in parental cells (Fig. 6A). HER3-silenced cells responded better to drug treatment compared to the control cells, whereas HER3-overexpressed cells showed more resistance to DDP therapy (Fig. 6B and 6C). These data verified that HER3 is important in DDP resistance in ovarian cancer cells.

To determine if HER3 inhibitor TX1-85-1 may increase DDP sensitivity in ovarian cancer cells, a drug combination test of TX1-85-1 and DDP in A2780-DDP cells was performed. The Chou-Talalay method was used to investigate the synergistic effect of DDP (0, 0.1, 0.3, 0.5, 0.7, 1, 3, 5, 7, 10µM) and TX1-85-1 (0, 0.3, 0.7, 1, 3, 7µM). The Combination Index (CI) values were shown in Fig. S5D. The Fraction Affected-combination index (Fa) showed the effects of the combination with various dosages of TX1-85-1 and DDP (Fig. 6D). A scatter plot was used to illustrate the synergistic effect of these two drugs, and the Fa-CI plot was practically lower than one, showing that these two drugs had a substantial synergistic effect (Fig. 6E). The synergistic effect suggested that HER3 inhibition boosted resistant cell sensitivity to DDP treatment. Based on the CI values, we chose two compounds with high combined consistency (DDP 7µM, TX1-85-1 3 and 7µM) for apoptosis analysis. The DDP and TX1-85-1 groups had considerably higher percentages of apoptotic cells than those treated with a single treatment (Fig. 6F). The similar findings were observed in OVCAR3-DDP cells (Figs. S5E-S5G), and TX1-85-1 reversed the cell resistance to DDP treatment. Hence, these data demonstrated that HER3 mediated DDP resistance, and HER3 inhibitor made the resistant cells more sensitivity to DDP treatment.

### CYLD knockdown promoted DDP resistance by inhibiting cell apoptosis through increasing Bcl-XL and inhibiting Bax, and by inducing drug efflux via upregulating ABCB1 expression

**Fig. 7:**
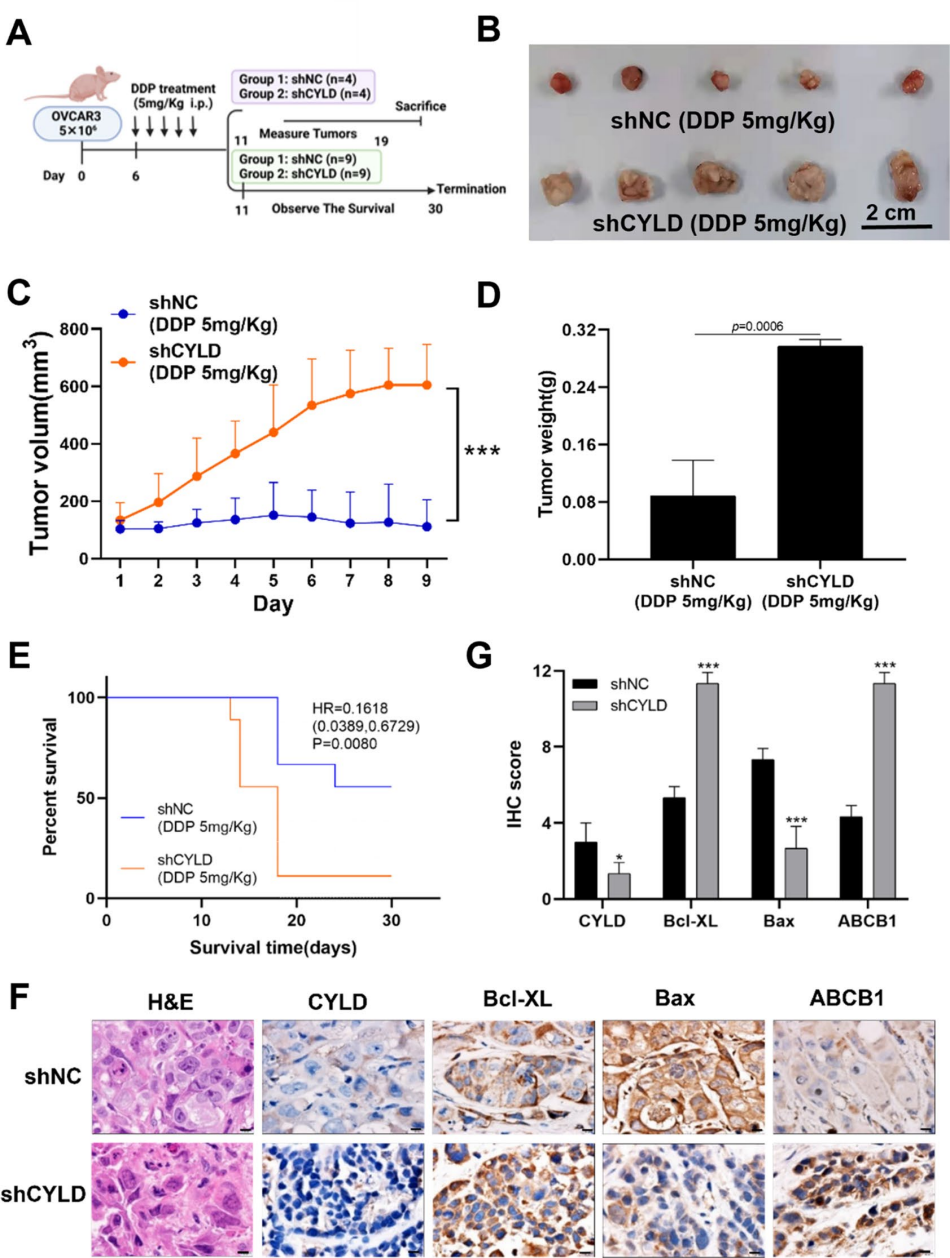
CYLD knockdown increased DDP resistance by inhibiting apoptosis and inducing ABCB1 expression in vivo (**A**,** B**). The shCYLD or shNC OVCAR3 cells were subcutaneously implanted into both sides of the armpit of BALB/C nude mice and treated with DDP (5 mg/Kg) intraperitoneally every day for 5 days, with tumor volumes assessed daily. The quantified results and graph were presented. (**C**,** D**,** E**). The tumor growth, tumor weight, and total survival curves were displayed. (**F**). IHC staining revealed the expression levels of CYLD, Bcl-XL, Bax, and ABCB1. (**G**). The quantifiable levels of staining data were represented by an IHC score and plotted in a histogram. Data were statistically analyzed with Student’s t-test and values are shown as mean ± SD. *, **, *** indicates significant difference at *p* < 0.05, *p* < 0.01, *p* < 0.001, respectively

In order to determine if CYLD knockdown enhances tumor growth and DDP resistance, we generated a xenograft model in BALB/C nude mice with OVCAR3-shNC and OVCAR3-shCYLD (Fig. 7A). The results indicated that CYLD knockdown greatly increased the tumor growth of OVCAR3 cells following DDP treatment (Fig. 7B and 7D). Furthermore, mice harboring OVCAR3-shCYLD cells had significantly shorter survival time than the control group (Fig. 7E). These data showed that CYLD knockdown increased DDP resistance of ovarian cancer cells and reduced survival time. The tumor tissues produced from the OVCAR3-shCYLD group had higher expression levels of Bcl-XL and ABCB1, but lower expression levels of Bax (Fig. 7F and 7G). Based on Bcl-XL and Bax being essential proteins in the apoptosis process, these results demonstrated that CYLD knockdown hindered the apoptotic pathway and elevated ABCB1 expression which may promote drug resistance through drug efflux in vivo.

### Forced expression of CYLD reversed DDP resistance, and dual treatment with DDP and ABCB1 inhibitors significantly inhibited tumorigenesis

**Fig. 8:**
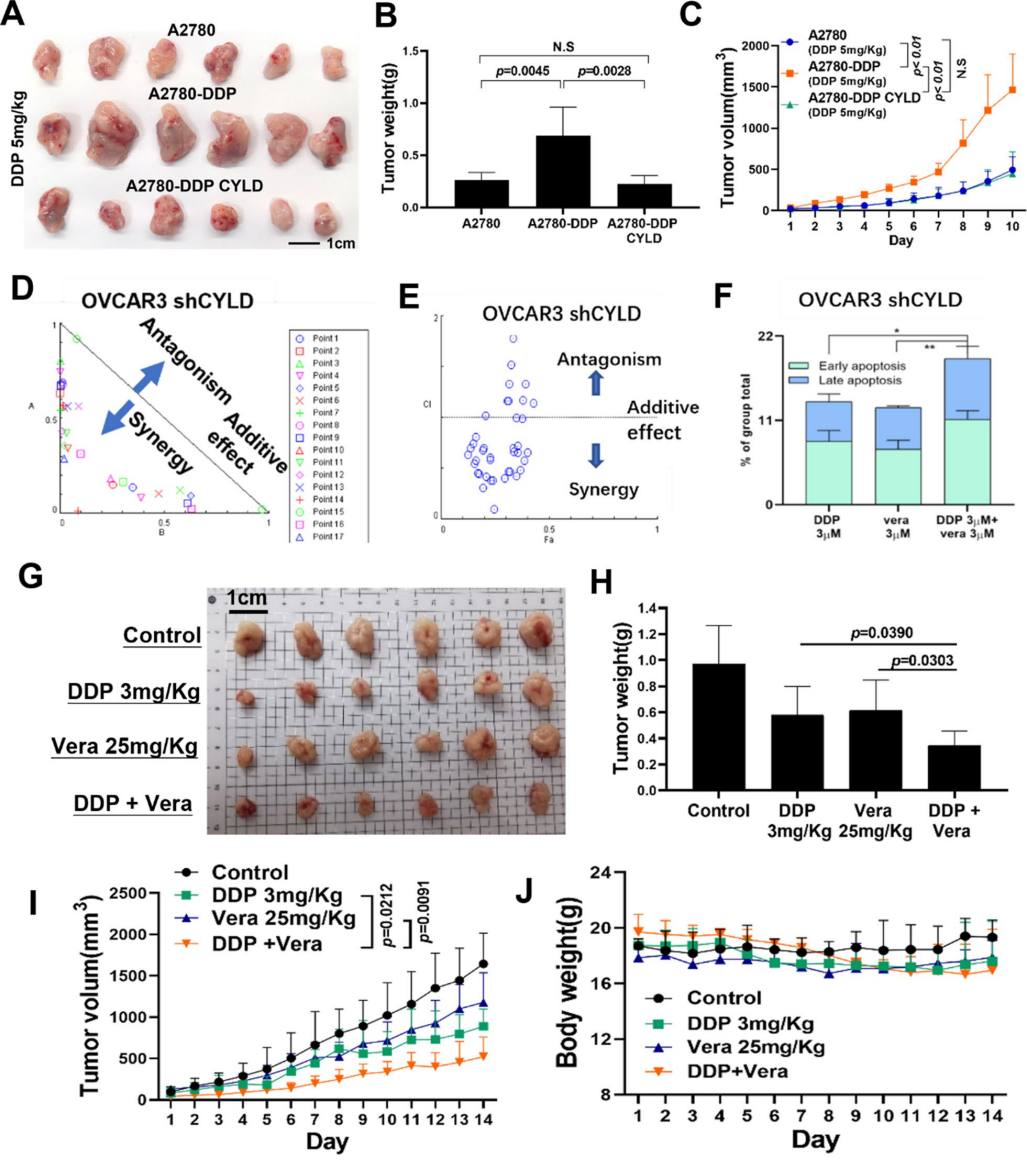
Forced expression of CYLD partially reversed DDP resistance, and combination treatment of DDP and ABCB1 inhibitors significantly inhibited tumorigenesis (**A**). Tumors from nude mice harboring A2780, A2780-DDP, and A2780-DDP with CYLD-overexpressed cells were observed. Tumor volumes were evaluated daily after 5 days of intraperitoneal administration of 5 mg/kg DDP. (**B**,** C**). The tumor development curve and tumor weight were shown by quantifiable dates and graphs. (**D**,** E**). The combination therapy analysis of DDP and Verapamil using CompuSyn software. The dose-effect curve and Fa-CI plots were displayed. DDP (3µM) and Verapamil (3µM) had a CI value of 0.3792. (**F**). Flow cytometry was used to determine the cell apoptosis rates of OVCAR3-shCYLD cells after co-treatment with DDP and Verapamil. (**G**). BALB/C nude mice were utilized to conduct a Xenograft experiment with OVCAR3 shCYLD cells, and tumor volumes were measured every day after treatment with DDP (3 mg/kg), Verapamil (25 mg/kg), or DDP (3 mg/kg) plus Verapamil (25 mg/kg) intraperitoneally for 5 days. (**H**,** I**,** J**). The tumor development curve, tumor weight, and animal weights were all measured and investigated. (**K**). A schematic graphic depicted that HER3/CYLD/ABCB1 is a novel signaling network that regulates tumor development and DDP resistance. Data were statistically analyzed with Student’s t-test and values are shown as mean ± SD. *, **, *** indicates significant difference at *p* < 0.05, *p* < 0.01, *p* < 0.001, respectively

Based on our earlier findings, we established a xenograft model of CYLD-overexpressed A2780-DDP cells and their control cells, A2780 and A2780-DDP (Fig. 8A). The results showed that CYLD overexpression significantly inhibited tumor growth in A2780-DDP cells after DDP treatment (Fig. 8B and 8C). These results showed that overexpressing CYLD partially reversed DDP resistance.

Then we investigated whether the ABCB1 inhibitor Verapamil may overcome DDP resistance triggered by CYLD knockdown. In CYLD knockdown OVCAR3 cells, synergistic effects of DDP (0, 0.1, 0.3, 0.5, 0.7, 1, 3, 5, 7, 10µM) and Verapamil (0, 1, 3, 7, 10, 30µM) were observed. The CI values for these two drugs were shown in Figure S5A. A scatter plot (Fig. 8D) and a Fa-CI plot (Fig. 8E) were used to characterize the synergistic effects of these two drugs in OVCAR3 cells. The CI results showed that DDP 3µM and Verapamil 3µM had the optimum combination effects, hence these dosages were chosen for further apoptosis experiment (Fig. S6B). The apoptotic rates of this combination group in OVCAR3 cells with CYLD knockdown were considerably higher than those of the single treatment (Fig. 8F). We also found similar results in A2780-shCYLD cells (Figs. S6C-S6F). To further test the synergistic effects of DDP and Verapamil, we constructed a xenograft model using OVCAR3-shCYLD cells. As shown in Fig. 8G and I, treatment with DDP or Verapamil alone did not effectively inhibit tumor progression, whereas the dual-treatment greatly inhibited tumor growth. In addition, the body weights were similar in all 4 groups, suggesting that the combination treatment at the indicated doses did not cause significant toxicity in mice (Fig. 8J).

### Combination treatment of DDP and Verapamil/TX1-85-1 significantly induced apoptosis in primary ovarian cancer cells and significantly inhibited tumorigenesis

**Fig. 9:**
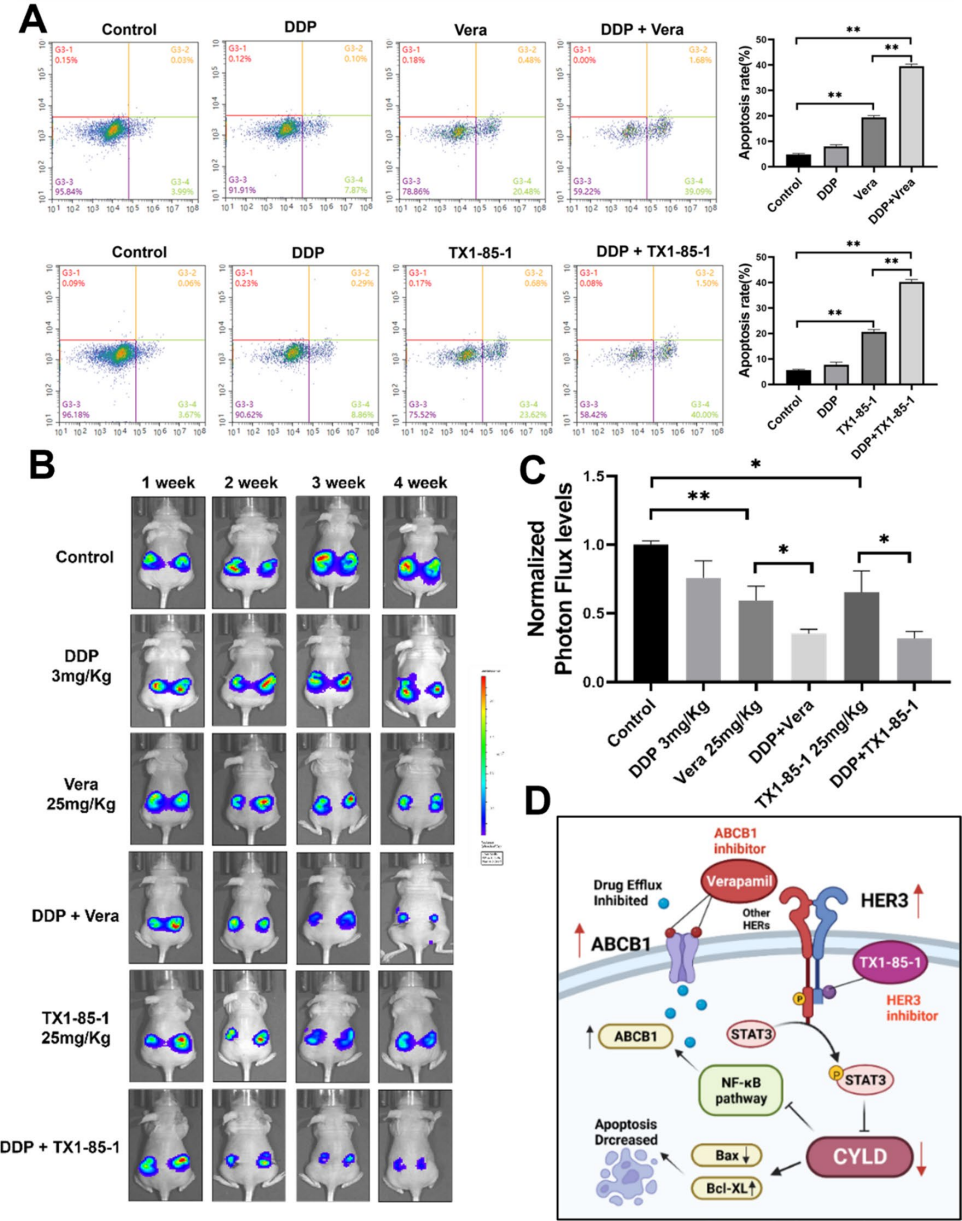
Combination treatment of DDP and Verapamil/TX1-85-1 significantly induced apoptosis rates of primary ovarian cancer cells, and inhibited tumor growth rates (**A**). Cellular apoptosis analysis was performed by using primary ovarian cancer cells, and combination treatment of DDP and TX1-85-1/Verapamil showed significantly higher percentages of apoptotic cells than those treated by a single treatment. (**B**,** C**) CDX (cell derived xenograft) model was constructed by injecting cancer cell lines carrying luciferase construct into the ovaries of nude mice. Cells were resuspended in 30 µL PBS mixed with Matrigel at a ratio of 1:1 and injected into nude mice via intrabursal injection of mouse ovary through the surgery. After about 1 week of cell injection, the mice were randomly divided into 6 treatment groups: control, DDP (3 mg/kg, intraperitoneally), Verapamil (25 mg/kg, intraperitoneally), DDP + Verapamil, TX1-85-1(25 mg/kg, intraperitoneally), DDP + TX1-85-1; then treated as indicated for 2 times a week. The tumor volumes were monitored and calculated by using luminescence intensity signals per sec using an IVIS Lumina III In Vivo Imaging System (PerkinElmer). (**D**) A schematic graphic depicted that HER3/CYLD/ABCB1 is a novel signaling network that regulates tumor development and DDP resistance. Data were statistically analyzed by using Student’s t-test and values are shown as mean ± SD. *, **indicate significant difference at *p* < 0.05, *p* < 0.01, respectivelsy

In this study, we cultured primary ovarian cancer cells from ovarian cancer tumor specimens and performed apoptosis analysis of the primary ovarian cancer cells with or without combination treatment of DDP and TX1-85-1/Verapamil, and showed that significant higher percentages of apoptotic cells were detected in the combination group compared to a single treatment (Fig. 9A). In addition, we conducted new experiment with CDX (cell derived xenograft) model constructed by injecting ovarian cancer cell lines carrying luciferase construct into the ovaries of nude mice. After about 1 week of cell injection, the mice were randomly divided into 6 treatment groups: control, DDP (3 mg/kg, intraperitoneally), Verapamil (25 mg/kg, intraperitoneally), DDP + Verapamil, TX1-85-1 (25 mg/kg, intraperitoneally), DDP + TX1-85-1; then treated for 2 times a week as indicated above. The tumor volumes were monitored and calculated as luminescence intensity signals per sec using an IVIS Lumina III In Vivo Imaging System. These results showed that dual-treatment of DDP + Verapamil/DDP + TX1-85-1 significantly decreased ovarian tumor growth compared to the single treatment (Fig. 9B and 9C). Taken together our findings demonstrated that CYLD was a DDP sensitizer which may be as a new biomarker for ovarian cancer DDP resistance, the HER3 inhibitor and ABCB1 inhibitor combination greatly increased cisplatin treatment effect, and made DDP resistant OC cells to become sensitive cells (Fig. 9D).

## Discussion

Platinum resistance is one of the major problems that significantly affects the prognosis of ovarian cancer patients [40]. The 5-year survival rate of platinum-resistant patients is around 30% [41], which is much lower than that of platinum-sensitive patients [2]. Understanding the molecular mechanisms and clinical aspects is critical in developing the appropriate combination therapy method for attaining the best clinical outcomes. Interestingly, we found that CYLD expression levels were significantly downregulated in cisplatin-resistant OC, which was associated with a poor prognosis. CYLD was proposed as a key regulator of carcinogenesis in a variety of malignancies, including gastric and lung cancer [23, 24, 36, 37]. We suggest that CYLD plays an essential role in platinum resistance in ovarian cancer. To test the concept, we conducted both gain- and loss-of-function experiments and found that forced CYLD expression reduced cisplatin resistance by inducing apoptosis and expression of apoptosis-related molecules. Overexpression of CYLD also reversed DDP resistance, causing cisplatin-resistant OC to become DDP sensitive in vivo. These findings suggested that CYLD may be used for the diagnosis or/and therapeutic target for DDP resistant ovarian cancer.

The platinum-free interval (PFI) for ovarian cancer is defined as the time between the patient’s last dose of platinum-based chemotherapy and disease progression that is less than 6 months [42]. Previous platinum resistance research primarily focused on apoptosis-related pathways, such as the production of apoptosis inducer molecules as “caspase” [43, 44] or apoptosis inhibitor molecules as “Bcl-2” [45, 46]. We found that HER3 expression levels were significantly higher in ovarian DDP resistant cells than the sensitive cells. HER3 had no intrinsic tyrosine kinase capabilities unless it underwent hetrodimerization to produce dimers such as HER2-HER3 and HER1(EGFR)-HER3 [47]. However, the role of HER3 in DDP resistance is not yet established. Our group showed higher expression levels of HER3 in ovarian cancer cells were due to higher ROS levels [17] which played an important role in tumor angiogenesis [16]. HER3 could trigger the PI3K/Akt, MEK/MAPK, Jak/Stat, and Src kinase pathways [19]. In the current investigation, we discovered that HER3 expression enhanced DDP resistance in ovarian cancer cells, which was similar with clinical observations from prior studies [48]. In addition, HER3 inhibited CYLD expression by increasing STAT3 phosphorylation, but not through the p38, c-Jun, or Erk pathways. Interestingly, we discovered that combination of HER3 inhibitor TX1-85-1 with DDP had a significant synergistic effect to induce apoptosis, and drug dual-treatment of DDP resistant cells made the cells significantly sensitive to DDP, indicating that HER3 may be a new biomarker as well as therapeutic target to overcome DDP resistance, and that HER3 inhibitors may be used to overcome DDP resistance in the future.

Unexpectedly, we discovered increase of drug efflux in CYLD knockdown ovarian cancer cells using flow cytometry, which had not been reported yet. Higher drug efflux may be a significant mechanism of chemo-resistance [49], thus we predicted that CYLD mediated platinum resistance by inhibiting drug efflux. We found that CYLD facilitated drug resistance by downregulating the expression of ABCB1, a downstream molecule. CYLD knockdown resulted in NF-κB p65 translocation to the nucleus and stimulated ABCB1 expression through transcriptional activation. NF-κB p65 plays Versatile function in inflammation and cancer [50]. In 2003, two similar experimental studies were published in *Nature* to demonstrate that CYLD directly bound to the proline-rich sequence of NEMO, a regulatory molecule of NF-κB (an IκB kinase-adaptor protein), recruiting CYLD into the IKK complex; through subsequent de-ubiquitination, the ubiquitination-mediated degradation of IκB was reduced, resulting to the release of the p50-p65 (encoded by RELA) dimer and the reduction of translocation of p65 into the nucleus [51, 52]. Ubiquitin modification plays key roles in the regulation of tumor immunotherapy resistance and may be used as potential therapeutic targets [53]. Ubiquitination can interact with O-GlcNAcylation and be involved in the process of cancer resistance [54]. Under normal circumstances, the NF-κB dimer (p50/p65) binds to the IκB protein in the cytoplasm and remains in an inhibited state. When cells are stimulated by TNFα, signal transduction activates the IKK complex. IKKβ within the IKK complex phosphorylates IκB, leading to its ubiquitination and degradation via the proteasome, allowing it to be translocated into the nucleus. CYLD inhibited the activity of the IKK complex by removing the K63 ubiquitin chains on NEMO, the regulatory subunit of the IKK complex. This reduced the phosphorylation of IκB by decreasing its degradation and subsequently reducing the nuclear translocation of the NF-κB dimer. We found there was strong synergistic effect of combination therapy (ABCB1 inhibitor Verapamil and DDP), suggesting CYLD’s important role in mediating drug resistance. Thus, this combination treatments may be used to improve the efficacy of DDP, especially in DDP resistant OC in the future.

## Conclusions

In summary, we identified CYLD as an important regulator for DDP resistance. CYLD mediates the DDP resistance by inducing apoptosis through Bcl/Bax pathway and by regulating drug efflux through ABCB1 expression. HER3 is upstream mediator for suppressing CYLD expression. The drug combination of either HER3 inhibitor or ABCB1 inhibitor with DDP showed significantly synergistic effect to reverse the DDP resistance. HER3, CYLD, and/or ABCB1 may be used as novel therapeutic target(s) to overcome DDP resistance or used as new biomarker(s) of the DDP resistance for diagnosis in the future. Our results provide new mechanism and targets to overcome DDP resistance of ovarian cancer cells, which warrants further clinical study in the future.

## Electronic supplementary material

Below is the link to the electronic supplementary material.

Supplementary Material 1

## Data Availability

No datasets were generated or analysed during the current study.

## Abbreviations

OC: Ovarian cancer
CYLD: Cylindromatosis
DDP: Cisplatin
IHC: Immunohistochemistry
CCK-8: Cell counting kit 8
TCGA: The Cancer Genome Atlas
GEO: Gene Expression Omnibus

## References

[1] Shen L, Xia M, Zhang Y, Luo H, Dong D, Sun L. Mitochondrial integration and ovarian cancer chemotherapy resistance. Exp Cell Res 2021, 401(2). 10.1016/j.yexcr.2021.112549 33640393

[2] Munoz-Galvan S, Carnero A. Targeting Cancer stem cells to overcome therapy resistance in ovarian Cancer. Cells 2020, 9(6). 10.3390/cells9061402 PMC7349391 32512891

[3] Lukanović D, Herzog M, Kobal B, Černe K. The contribution of copper efflux transporters ATP7A and ATP7B to chemoresistance and personalized medicine in ovarian cancer. Biomed Pharmacother 2020, 129. 10.1016/j.biopha.2020.110401 32570116

[4] Kroeger PT Jr., Drapkin R. Pathogenesis and heterogeneity of ovarian cancer. Curr Opin Obstet Gynecol. 2017;29(1):26–34. 27898521 10.1097/GCO.0000000000000340 PMC5201412

[5] Daly MB, Pilarski R, Yurgelun MB, Berry MP, Buys SS, Dickson P, Domchek SM, Elkhanany A, Friedman S, Garber JE, et al. NCCN guidelines insights: Genetic/Familial High-Risk assessment: breast, ovarian, and pancreatic, version 1.2020. J Natl Compr Canc Netw. 2020;18(4):380–91. 32259785 10.6004/jnccn.2020.0017

[6] Grunewald T, Ledermann JA. Targeted therapies for ovarian Cancer. Best Pract Res Clin Obstet Gynecol. 2017;41:139–52. 10.1016/j.bpobgyn.2016.12.001 28111228

[7] Li T, Zhang H, Lian M, He Q, Lv M, Zhai L, Zhou J, Wu K, Yi M. Global status and attributable risk factors of breast, cervical, ovarian, and uterine cancers from 1990 to 2021. J Hematol Oncol. 2025;18(1):5. 39794860 10.1186/s13045-025-01660-y PMC11721161

[8] Ghosh SJBC. Cisplatin: The First Metal Based Anticancer Drug. 2019, 88:102925. 10.1016/j.bioorg.2019.102925 31003078

[9] Galluzzi L, Senovilla L, Vitale I, Michels J, Kroemer GJO. Molecular mechanisms of cisplatin resistance. 2012, 31(15):1869. 10.1038/onc.2011.384 21892204

[10] Tchounwou PB, Dasari S, Noubissi FK, Ray P. Kumar SJJoEP: advances in our Understanding of the molecular mechanisms of action of cisplatin in Cancer therapy. 2021, 13:303–28. 10.2147/JEP.S267383 PMC7987268 33776489

[11] Freimund AE, Beach JA, Christie EL, Bowtell DJHOCNA. Mechanisms of drug resistance in High-Grade serous ovarian Cancer. 2018, 32(6):983–96. 10.1016/j.hoc.2018.07.007 30390769

[12] Khan MA, Vikramdeo KS, Sudan SK, Singh S, Wilhite A, Dasgupta S, Rocconi RP, Singh AP. Platinum-resistant ovarian cancer: from drug resistance mechanisms to liquid biopsy-based biomarkers for disease management. Semin Cancer Biol. 2021;77:99–109. 34418576 10.1016/j.semcancer.2021.08.005 PMC8665066

[13] Wu C, He L, Wei Q, Li Q, Jiang L, Zhao L, Wang C, Li J, Wei M. Bioinformatic profiling identifies a platinum-resistant-related risk signature for ovarian cancer. Cancer Med. 2020;9(3):1242–53. 31856408 10.1002/cam4.2692 PMC6997076

[14] Haikala HM, Janne, PAJCcraojotAAfCR. Thirty Years of HER3: From Basic Biology to Therapeutic Interventions. 27(13):3528–3539. 10.1158/1078-0432.CCR-20-4465 PMC8254743 33608318

[15] Kiavue N, Cabel L, Melaabi S, Bataillon G, Callens C, Lerebours F, Pierga JY, Bidard FC. ERBB3 mutations in cancer: biological aspects, prevalence and therapeutics. Oncogene. 2020;39(3):487–502. 31519989 10.1038/s41388-019-1001-5

[16] He J, Jing Y, Li W, Qian X, Xu Q, Li FS, Liu LZ, Jiang BH, Jiang Y. Roles and mechanism of miR-199a and miR-125b in tumor angiogenesis. PLoS ONE. 2013;8(2):e56647. 23437196 10.1371/journal.pone.0056647 PMC3577861

[17] He J, Xu Q, Jing Y, Agani F, Qian X, Carpenter R, Li Q, Wang XR, Peiper SS, Lu Z, et al. Reactive oxygen species regulate ERBB2 and ERBB3 expression via miR-199a/125b and DNA methylation. EMBO Rep. 2012;13(12):1116–22. 23146892 10.1038/embor.2012.162 PMC3512405

[18] Yonesaka K. HER2-/HER3-Targeting Antibody-Drug conjugates for treating lung and colorectal cancers resistant to EGFR inhibitors. Cancers (Basel) 2021, 13(5). 10.3390/cancers13051047 PMC7958627 33801379

[19] Liu X, Liu S, Lyu H, Riker AI, Zhang Y, Liu B. Development of effective therapeutics targeting HER3 for Cancer treatment. Biol Proced Online. 2019;21:5. 30930695 10.1186/s12575-019-0093-1 PMC6425631

[20] Cui Z, Kang H, Grandis JR, Johnson DE. CYLD alterations in the tumorigenesis and progression of human Papillomavirus-Associated head and neck cancers. Mol Cancer Res. 2021;19(1):14–24. 32883697 10.1158/1541-7786.MCR-20-0565 PMC7840145

[21] Yang Y, Zhou J. CYLD - a deubiquitylase that acts to fine-tune microtubule properties and functions. J Cell Sci. 2016;129(12):2289–95. 27173491 10.1242/jcs.183319

[22] Lork M, Verhelst K, Beyaert R. CYLD, A20 and OTULIN deubiquitinases in NF-kappaB signaling and cell death: so similar, yet so different. Cell Death Differ. 2017;24(7):1172–83. 28362430 10.1038/cdd.2017.46 PMC5520167

[23] Suenaga N, Kuramitsu M, Komure K, Kanemaru A, Jono HJIJMS. Loss of tumor suppressor CYLD expression triggers cisplatin resistance in oral squamous cell carcinoma. 2019, 20(20):5194-. 10.3390/ijms20205194 PMC6829433 31635163

[24] Zhao J, Wang X, Mi Z, Jiang X, Sun L, Zheng B, Wang J, Meng M, Zhang L, Wang Z, et al. STAT3/miR-135b/NF-kappaB axis confers aggressiveness and unfavorable prognosis in non-small-cell lung cancer. Cell Death Dis. 2021;12(5):493. 33990540 10.1038/s41419-021-03773-x PMC8121828

[25] Delbridge AR, Valente LJ, Strasser A. The role of the apoptotic machinery in tumor suppression. Cold Spring Harb Perspect Biol 2012, 4(11). 10.1101/cshperspect.a008789 PMC3536334 23125015

[26] Dondelinger Y, Darding M, Bertrand MJ, Walczak H. Poly-ubiquitination in TNFR1-mediated necroptosis. Cell Mol Life Sci. 2016;73(11–12):2165–76. 27066894 10.1007/s00018-016-2191-4 PMC4887548

[27] Liu WJ, Huang YX, Wang W, Zhang Y, Liu BJ, Qiu JG, Jiang BH, Liu LZ. NOX4 signaling mediates Cancer development and therapeutic resistance through HER3 in ovarian Cancer cells. Cells 2021, 10(7). 10.3390/cells10071647 PMC8304464 34209278

[28] Prat J. FIGO’s staging classification for cancer of the ovary, fallopian tube, and peritoneum: abridged republication. J Gynecologic Oncol 2015, 26(2). 10.3802/jgo.2015.26.2.87 PMC4397237 25872889

[29] Meinhold-Heerlein I, Fotopoulou C, Harter P, Kurzeder C, Mustea A, Wimberger P, Hauptmann S, Sehouli J. The new WHO classification of ovarian, fallopian tube, and primary peritoneal cancer and its clinical implications. Arch Gynecol Obstet. 2016;293(4):695–700. 26894303 10.1007/s00404-016-4035-8

[30] Werner B, Yuwono N, Duggan J, Liu D, David C, Srirangan S, Provan P, Investigators IN, DeFazio A, Arora V, et al. Cell-free DNA is abundant in Ascites and represents a liquid biopsy of ovarian cancer. Gynecol Oncol. 2021;162(3):720–7. 34454680 10.1016/j.ygyno.2021.06.028

[31] Ma H-M, Cui N, Zheng P-S. HOXA5 inhibits the proliferation and neoplasia of cervical cancer cells via downregulating the activity of the Wnt/β-catenin pathway and transactivating TP53. Cell Death Dis 2020, 11(6). 10.1038/s41419-020-2629-3 PMC7272418 32499530

[32] Qiu J, Li Q, Bell KA, Yao X, Du Y, Zhang E, Yu JJ, Yu Y, Shi Z, Jiang J. Small-molecule Inhibition of prostaglandin E receptor 2 impairs cyclooxygenase‐associated malignant glioma growth. Br J Pharmacol. 2019;176(11):1680–99. 30761522 10.1111/bph.14622 PMC6514294

[33] Xia Z-K, Wang W, Qiu J-G, Shi X-N, Li H-J, Chen R, Ke K-B, Dong C, Zhu Y, Wu S-G et al. Discovery of a new CDK4/6 and PI3K/AKT multiple kinase inhibitor aminoquinol for the treatment of hepatocellular carcinoma. Front Pharmacol 2021, 12. 10.3389/fphar.2021.691769 PMC8320333 34335258

[34] Hu J, Meng Y, Zhang Z, Yan Q, Jiang X, Lv Z, Hu L. MARCH5 RNA promotes autophagy, migration, and invasion of ovarian cancer cells. Autophagy. 2016;13(2):333–44. 27875077 10.1080/15548627.2016.1256520 PMC5324849

[35] Agarwal S, Liang G, Ahlqvist K, Pannem R, Posern G, Massoumi R. Serum response factor controls CYLD expression via MAPK signaling pathway. PLoS ONE 2011, 6(5). 10.1371/journal.pone.0019613 PMC3088714 21573132

[36] Welte S, Urbanik T, Elssner C, Kautz N, Koehler BC, Waldburger N, Bermejo JL, Pinna F, Weiss KH, Schemmer P, et al. Nuclear expression of the deubiquitinase CYLD is associated with improved survival in human hepatocellular carcinoma. PLoS ONE. 2014;9(10):e110591. 25329885 10.1371/journal.pone.0110591 PMC4199737

[37] Wang Z, Wang Q, Xu G, Meng N, Huang X, Jiang Z, Chen C, Zhang Y, Chen J, Li A, et al. The long noncoding RNA CRAL reverses cisplatin resistance via the miR-505/CYLD/AKT axis in human gastric cancer cells. RNA Biol. 2020;17(11):1576–89. 31885317 10.1080/15476286.2019.1709296 PMC7567514

[38] Stieg DC, Wang Y, Liu L-Z, Jiang B-H. ROS and MiRNA dysregulation in ovarian Cancer development, angiogenesis and therapeutic resistance. Int J Mol Sci 2022, 23(12). 10.3390/ijms23126702 PMC9223852 35743145

[39] Zeng H, Wang W, Zhang L, Lin Z. HER3-targeted therapy: the mechanism of drug resistance and the development of anticancer drugs. Cancer Drug Resist 2024. 10.20517/cdr.2024.11 PMC11149107 38835349

[40] He J, Yu JJ, Xu Q, Wang L, Zheng JZ, Liu LZ, Jiang BH. Downregulation of ATG14 by EGR1-MIR152 sensitizes ovarian cancer cells to cisplatin-induced apoptosis by inhibiting cyto-protective autophagy. Autophagy. 2015;11(2):373–84. 25650716 10.1080/15548627.2015.1009781 PMC4502709

[41] Hao L, Wang JM, Liu BQ, Yan J, Li C, Jiang JY, Zhao FY, Qiao HY, Wang HQ. m6A-YTHDF1-mediated TRIM29 upregulation facilitates the stem cell-like phenotype of cisplatin-resistant ovarian cancer cells. Biochim Biophys Acta Mol Cell Res. 2021;1868(1):118878. 33011193 10.1016/j.bbamcr.2020.118878

[42] Kumar S, Oien DB, Khurana A, Cliby W, Hartmann L, Chien J, Shridhar V. Coiled-Coil and C2 Domain-Containing protein 1A (CC2D1A) promotes chemotherapy resistance in ovarian Cancer. Front Oncol. 2019;9:986. 31632917 10.3389/fonc.2019.00986 PMC6779793

[43] Janson V, Johansson A, Grankvist K. Resistance to caspase-8 and– 9 fragments in a malignant pleural mesothelioma cell line with acquired cisplatin-resistance. Cell Death Dis. 2010;1:e78. 21364680 10.1038/cddis.2010.54 PMC3032340

[44] Cui Z, Pu T, Zhang Y, Wang J, Zhao Y. Long non-coding RNA LINC00346 contributes to cisplatin resistance in nasopharyngeal carcinoma by repressing miR-342-5p. Open Biol. 2020;10(5):190286. 32397872 10.1098/rsob.190286 PMC7276527

[45] Long X, Xiong W, Zeng X, Qi L, Cai Y, Mo M, Jiang H, Zhu B, Chen Z, Li Y. Cancer-associated fibroblasts promote cisplatin resistance in bladder cancer cells by increasing IGF-1/ERbeta/Bcl-2 signalling. Cell Death Dis. 2019;10(5):375. 31076571 10.1038/s41419-019-1581-6 PMC6510780

[46] Kuo KL, Liu SH, Lin WC, Hsu FS, Chow PM, Chang YW, Yang SP, Shi CS, Hsu CH, Liao SM et al. Trifluoperazine, an antipsychotic drug, effectively reduces drug resistance in Cisplatin-Resistant urothelial carcinoma cells via suppressing Bcl-xL: an in vitro and in vivo study. Int J Mol Sci 2019, 20(13). 10.3390/ijms20133218 PMC6651283 31262032

[47] Molavipordanjani S, Hosseinimehr SJ. The radiolabeled HER3 targeting molecules for tumor imaging. Iran J Pharm Res. 2021;20(1):141–52. 34400948 10.22037/ijpr.2021.114677.14991 PMC8170765

[48] McEvoy LM, O’Toole SA, Spillane CD, Martin CM, Gallagher MF, Stordal B, Blackshields G, Sheils O, O’Leary JJ. Identifying novel hypoxia-associated markers of chemoresistance in ovarian cancer. BMC Cancer. 2015;15:547. 26205780 10.1186/s12885-015-1539-8 PMC4513971

[49] Zhai J, Shen J, Xie G, Wu J, He M, Gao L, Zhang Y, Yao X, Shen L. Cancer-associated fibroblasts-derived IL-8 mediates resistance to cisplatin in human gastric cancer. Cancer Lett. 2019;454:37–43. 30978440 10.1016/j.canlet.2019.04.002

[50] Ma Q, Hao S, Hong W, Tergaonkar V, Sethi G, Tian Y, Duan C. Versatile function of NF-ĸB in inflammation and cancer. Exp Hematol Oncol. 2024;13(1):68. 39014491 10.1186/s40164-024-00529-z PMC11251119

[51] Brummelkamp TR, Nijman SM, Dirac AM, Bernards RJN. Loss of the cylindromatosis tumour suppressor inhibits apoptosis by activating NF-kappaB. 2003, 424(6950):797–801. 10.1038/nature01811 12917690

[52] Eirini T. Eudoxia, Hatzivassiliou, Theodore, Tsichritzis, Hannah, Farmer, Alan, Nature AJ: CYLD is a deubiquitinating enzyme that negatively regulates NF-κB activation by TNFR family members. 2003, 424(6950). 10.1038/nature01803 12917689

[53] Hong Z, Liu F, Zhang Z. Ubiquitin modification in the regulation of tumor immunotherapy resistance mechanisms and potential therapeutic targets. Exp Hematol Oncol. 2024;13(1):91. 39223632 10.1186/s40164-024-00552-0 PMC11367865

[54] Sun K, Zhi Y, Ren W, Li S, Zheng J, Gao L, Zhi K. Crosstalk between O-GlcNAcylation and ubiquitination: a novel strategy for overcoming cancer therapeutic resistance. Exp Hematol Oncol. 2024;13(1):107. 39487556 10.1186/s40164-024-00569-5 PMC11529444

