# Supplementary material for: Suppression of CYLD by HER3 confers ovarian cancer platinum resistance via inhibiting apoptosis and by inducing drug efflux
Source: Exp Hematol Oncol. 2025 Feb 26;14:21. doi: 10.1186/s40164-025-00620-z (PMC11866804; doi:10.1186/s40164-025-00620-z)
Supplement: Supplementary file 1 — Supplementary Material 1 [file 40164_2025_620_MOESM1_ESM.docx]

**Supplemental Figure legends**

**Sup.Figure.1 CYLD expression was downregulated in several cancers and associated with ovarian cancer clinical stage and prognosis.**

(A). The CYLD expression levels were analyzed in tumor and normal tissues of 33 human cancers by pan-cancer analysis through UCSC Xena database (<https://xenabrowser.net/datapages/>). **, ***indicates significant difference at p< 0.01 and p< 0.001, respectively. (B). The protein expression levels of CYLD in the ovarian cancer tissues were analyzed by using CPTAC database. **indicates significant difference at p< 0.01.

**
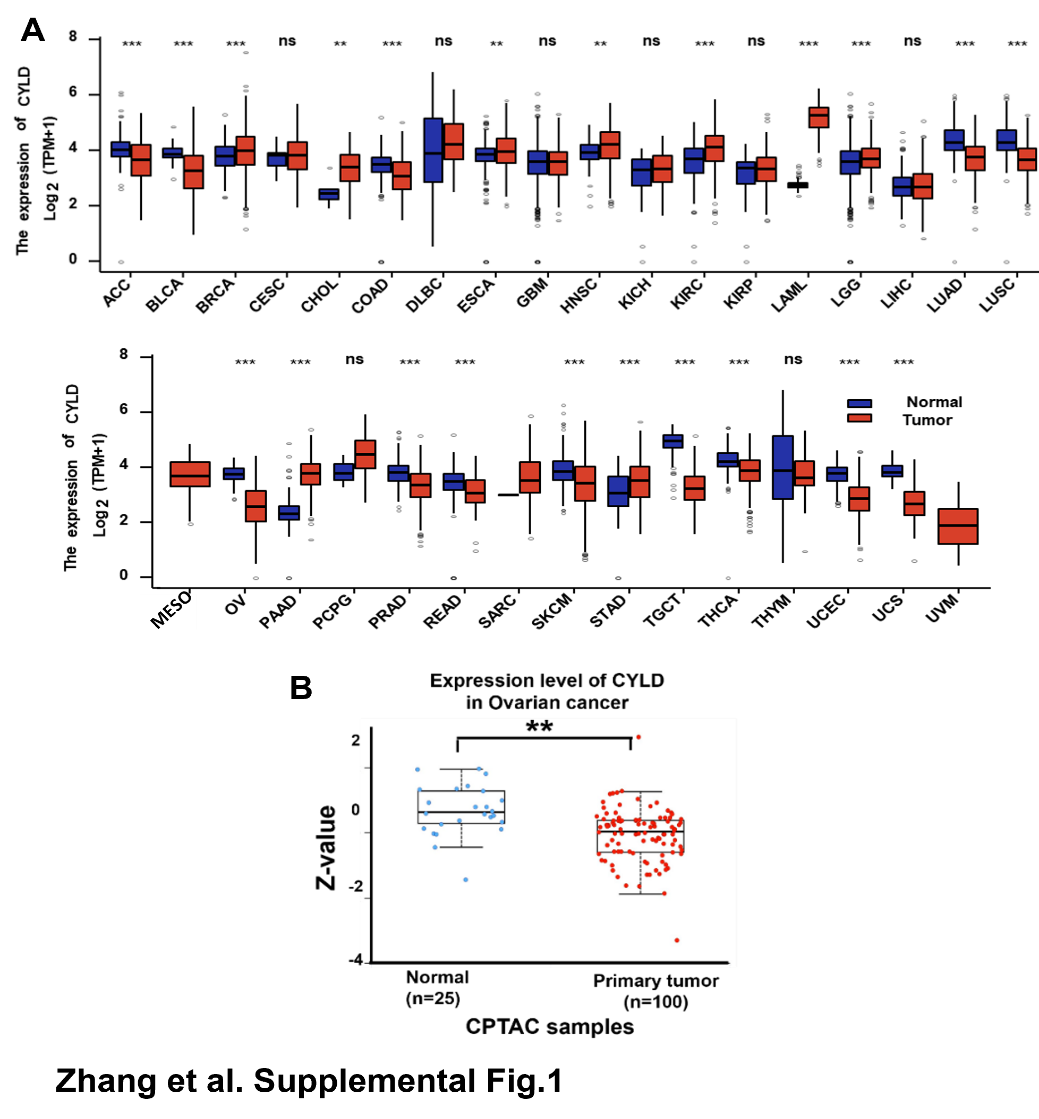
**

**Sup.Figure.2** **CYLD knockdown inhibited apoptosis activity rates in ovarian cancer cells.**

(A). GSEA analysis in ovarian cancer samples from the GEO dataset (GSE9891) showed that higher CYLD expression was positively correlated with apoptosis signaling. (B, C). Incubated with 5μM DDP or PBS for 48h, the apoptosis rates of CYLD knockdown OVCAR3/A2780 cells and control cells were detected by Flow cytometry, and the apoptosis rates were illustrated by the percentage sum of early and late apoptosis. (D). The apoptosis rates of A2780/A2780-DDP/A2780-DDP with CYLD overexpressed cell lines, after incubated with DDP for 48h. (E). We conducted Western blotting to test whether CYLD regulated DDP sensitivity by modulating other cell death associated molecules. CYLD did not influence the expression levels of RIP3, p62 and ATG5, as well as levels of GPX4 and SLC7A11in ovarian cancer A2780 and OVCAR3 cell lines. Data were statistically analyzed with Student’s t-test and values were shown as mean ± SD. *indicates significant difference at p < 0.05.


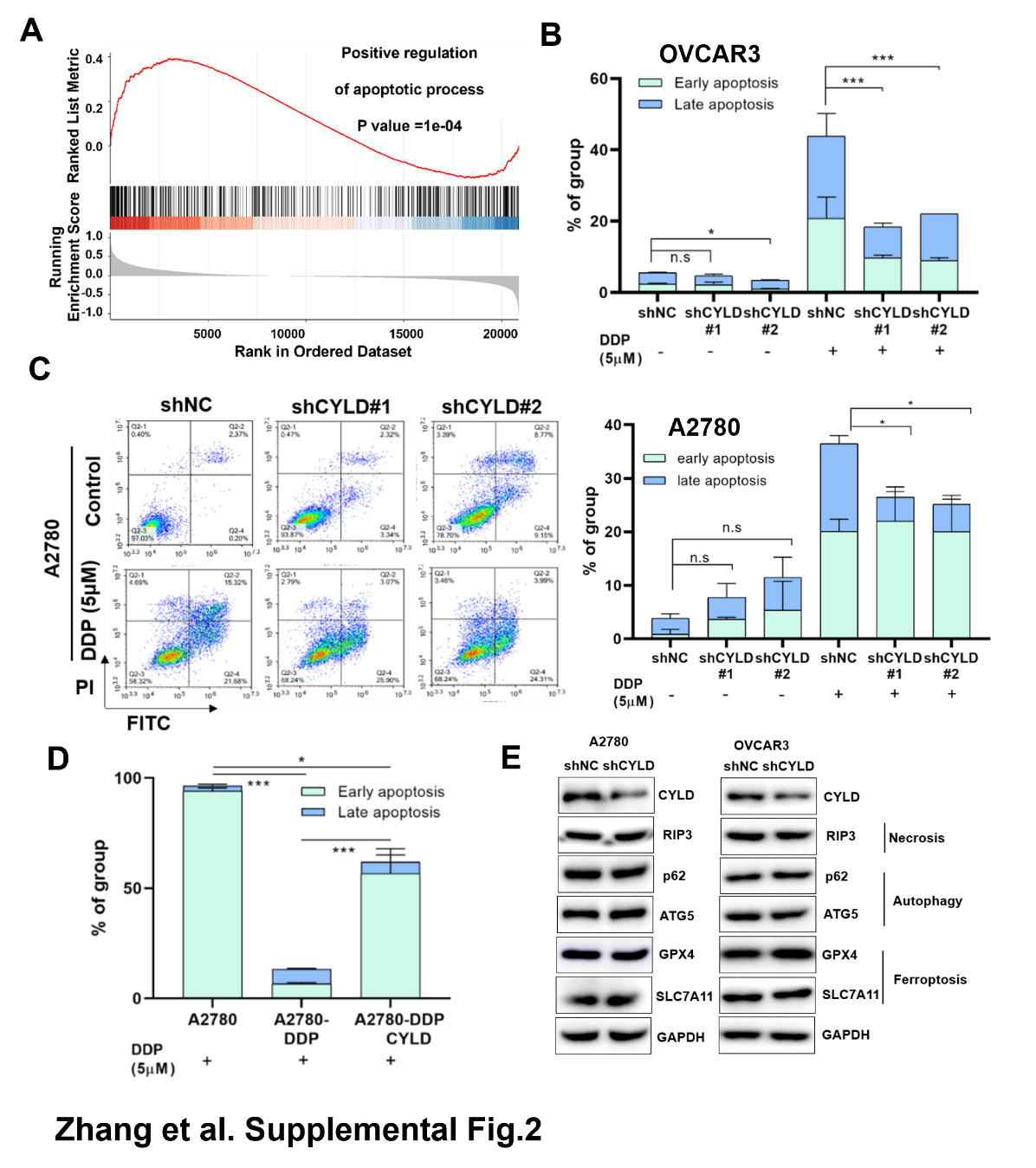


**Sup. Figure. 3 CYLD knockdown induced DDP resistance via promoting the expression of drug pump protein ABCB1.**

(A, B). The expression levels of ABC family proteins (ABCC1, ABCG and ABCG9) in OVCAR3-shCYLD cells and its control cells were detected by Western blotting and qRT-PCR. (C). After overexpression of CYLD in HEK293T cells, ABCB1 protein was immunoprecipitated using CYLD antibody, then detected and analyzed by Western blotting as indicated. (D). The relationship between the expression levels of ABCB1 and CYLD was analyzed in ovarian cancer samples. (E). CYLD knockdown induced translocation of p65 into the nucleus of OVCAR3 cells analyzed by immunofluorescence. (F, G). Inhibition of CYLD promoted transcriptional activities of NF-κB, which was normalized by Dual-Luciferase reporter activity. Data were statistically analyzed with Student’s t-test and values were shown as mean ± SD. *, **, ***indicates significant difference at p< 0.05, p< 0.01 and p< 0.001, respectively.


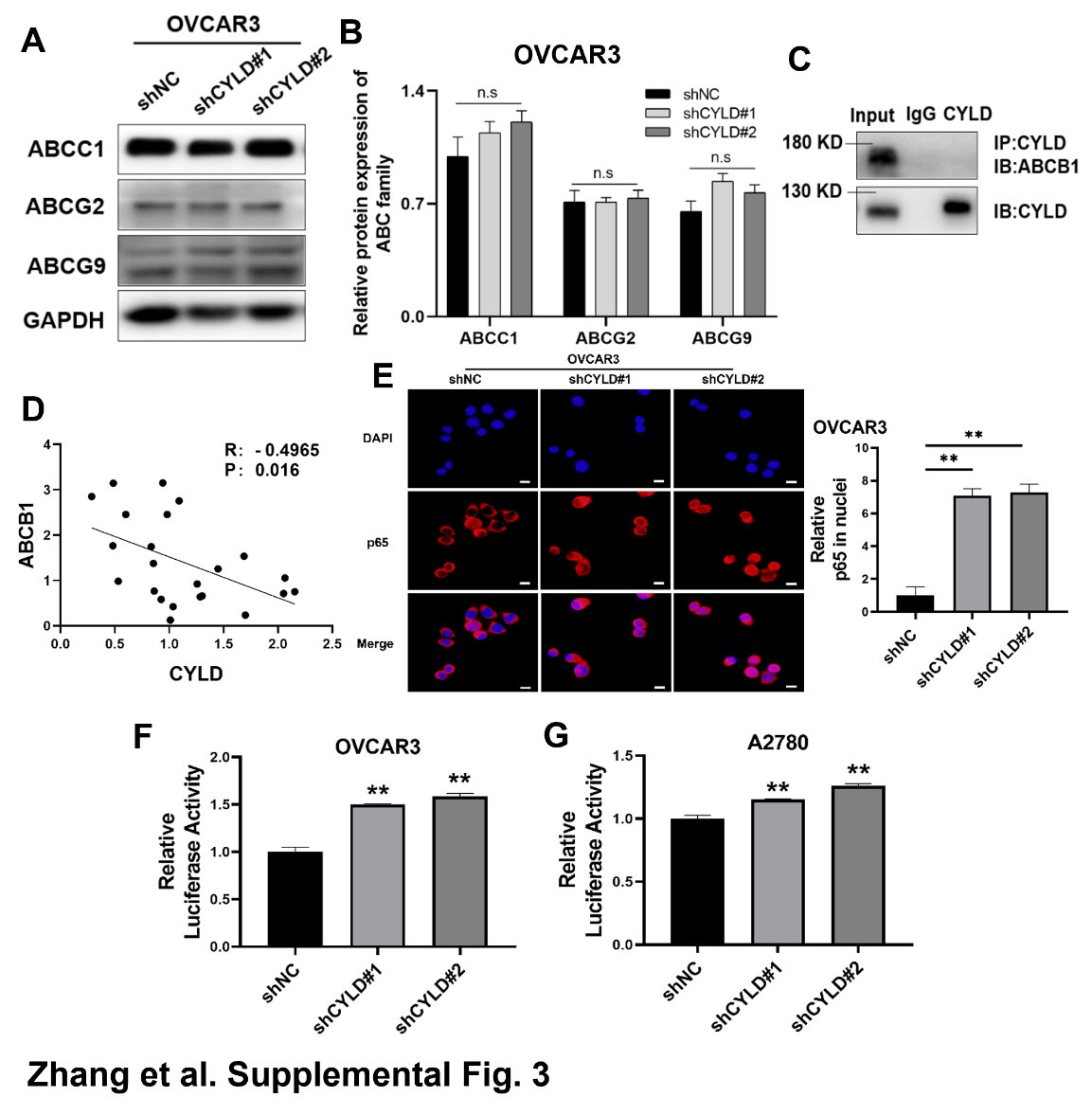


**Sup. Figure. 4 The p65 was a transcriptional activator of ABCB1 .**

(A). The base sequence of the potential binding of p65 with ABCB1 promoter was predicted by Jaspar website. (B). ABCB1 wild-type (WT) or mutant (Mut), which sequence was changed as indicated, reporter plasmids were constructed and co-transfected with control or p65 overexpression plasmid into 293T cells, and luciferase activities were analyzed and normalized to the mean value of control.

**
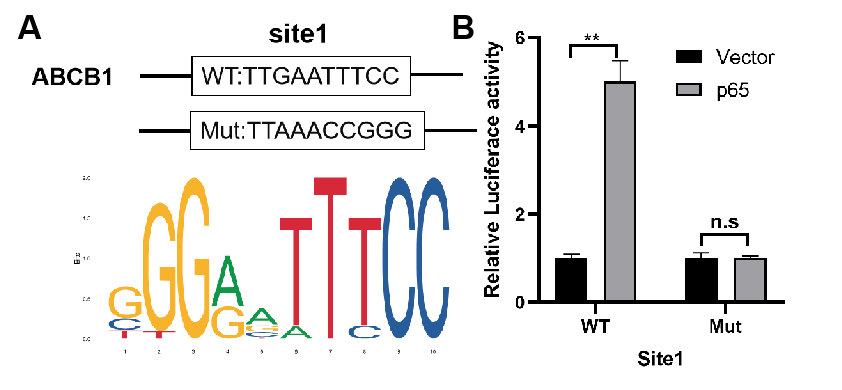
**

**Sup. Figure. 5 HER3 mediated cisplatin resistance and HER3 inhibitor rendered ovarian cancer cells more sensitive to cisplatin.**

(A). The correlations of CYLD and other three ERBB family members (EGFR, HER2, HER4) were measured by Spearman correlation in ovarian cancer patient samples. (B). Western blot analysis showed that protein levels of p-p38, p-Erk and p-c-Jun in HER3 overexpressed OVCAR3/A2780 cells. (C). STAT3 knockdown in A2780 and OVCAR3 cells induced CYLD expression. We then treated A2780 and OVCAR3 cells with STAT3 inhibitor STAT3-IN-1 for 48h, and showed that inhibition of STAT3 increased CYLD expression levels. (D). The combination index (CI) data of different doses of DDP and TX1-85-1 co-treated A2780-DDP were analyzed by CompuSyn software. (E). The cellular apoptosis rates of cisplatin resistance cell lines (OVCAR3-DDP) co-treated by DDP and TX1-85-1 were detected by Flow Cytometry analysis. (F). And the apoptosis rate of OVCAR3-DDP were illustrated by the percentage sum of early and late apoptosis. (G). The drug combination analysis of DDP and TX1-85-1 in OVCAR3-DDP cell were analyzed using CompuSyn software. The Fa-CI plot was shown. Data were statistically analyzed with Student’s t-test and values were shown as mean ± SD. *indicates significant difference at p< 0.05.


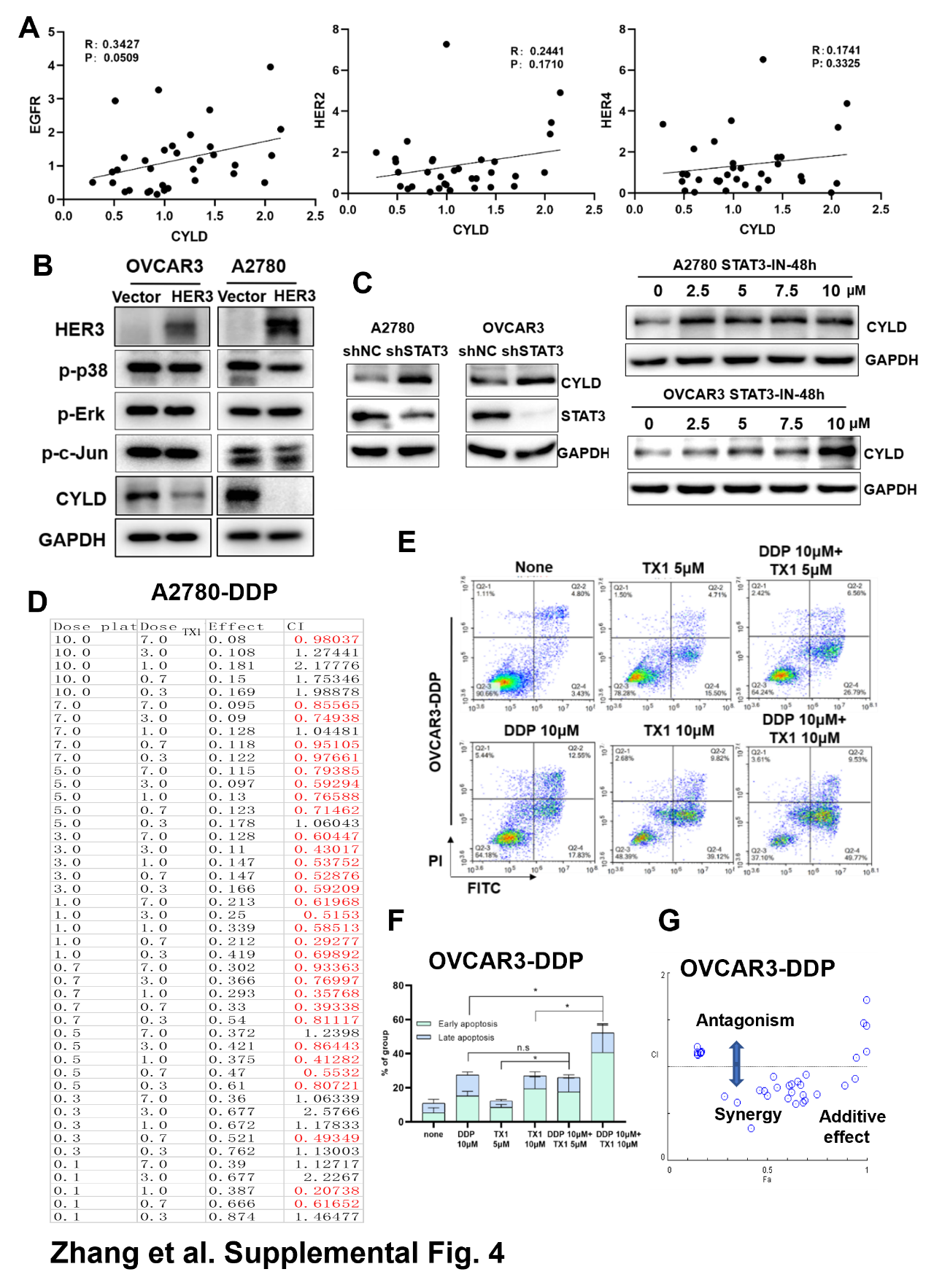


**Sup. Figure. 6 ABCB1 inhibitor made cisplatin-resistant cells to become sensitive.**

(A). The CI data of different doses of DDP and Verapamil co-treated OVCAR3-shCYLD were analyzed by CompuSyn software. (B, C). The cell apoptosis of cisplatin resistance cell lines co-treated by DDP and Verapamil were detected by Flow Cytometry analysis. (D). The apoptosis rates of A2780-shCYLD#1 were illustrated by the percentage sum of early and late apoptosis. (E, F). The drug combination analysis of DDP and Verapamil in A2780-shCYLD#1 cells were analyzed using CompuSyn software. The dose-effect curve and Fa-CI plot were shown. Data were statistically analyzed with Student’s t-test and values were shown as mean ± SD. *, **indicates significant difference at p< 0.05 and p< 0.01, respectively.


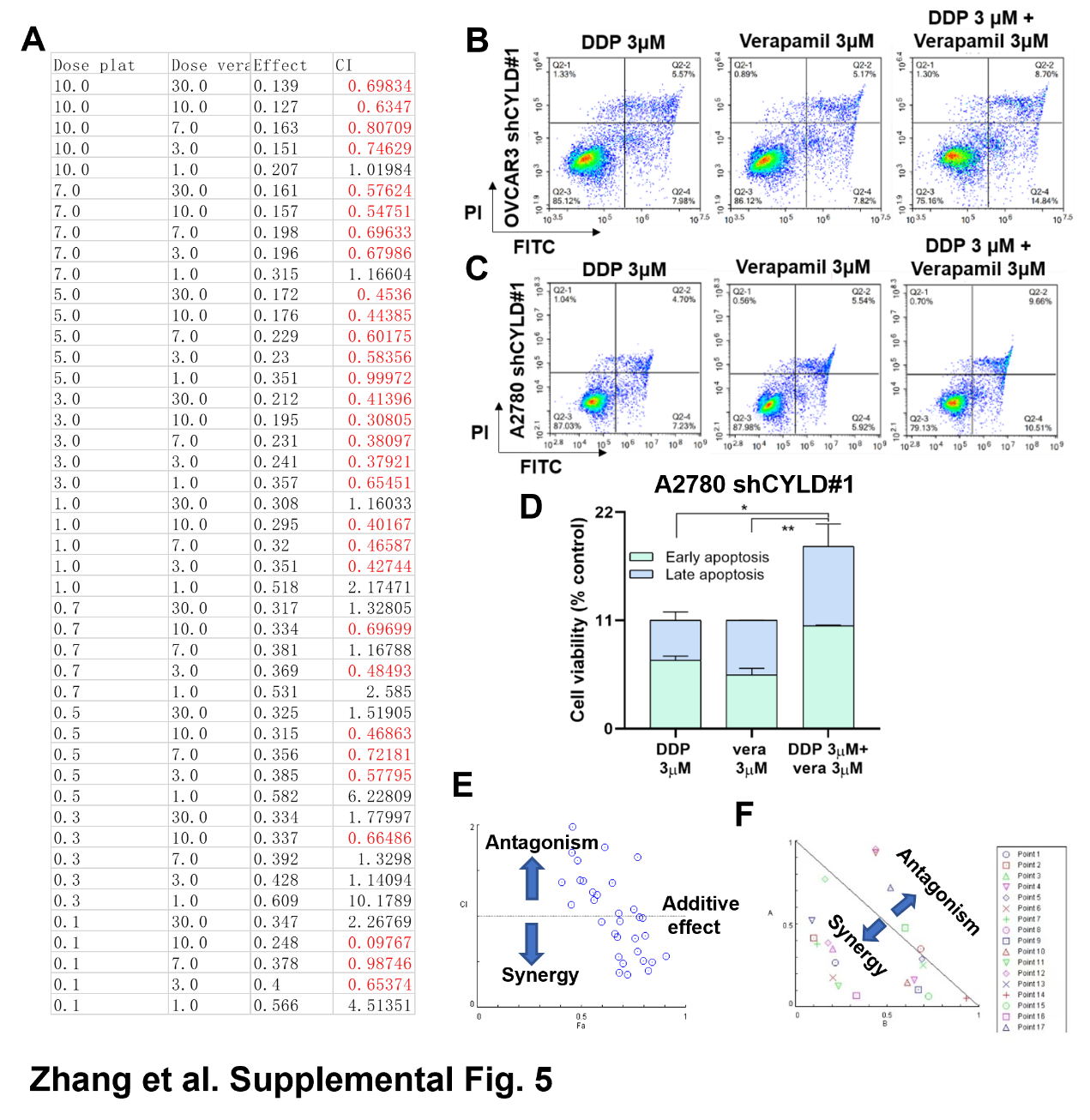


**Supplemental Tables**

**Sup. Table 1.** **Clinicopathological characteristics and the associations with CYLD expression in ovarian cancer tissues.**

| Clinicopathological Features | Cas No. | CYLD expression level | | *p* value |
| --- | --- | --- | --- | --- |
|  |  | Low (n=19) | High（n=14） |  |
| Age(year) |  |  |  |  |
| ＜55 | 19 | 11（33.33%） | 8（24.24%） | 0.6412 |
| ＞55 | 14 | 8（24.24%） | 6（18.18%） |  |
| Histological type |  |  |  |  |
| Serous | 30 | 18（54.55%） | 12（36.36%） | 0.7314 |
| Endometrioid | 2 | 0（0%） | 2（6.06%） |  |
| Clear cell | 1 | 1（3.03%） | 0（0%） |  |
| Ascites volume |  |  |  |  |
| ＜1000ml | 15 | 8（24.24%） | 7（21.21%） | 0.1286 |
| ＞1000ml | 18 | 11（33.33%） | 7（21.21%） |  |
| Epigastric metastasis |  |  |  |  |
| Yes | 23 | 13（39.39%） | 10（30.30%） | 0.3367 |
| No | 10 | 6（18.18%） | 4（12.12%） |  |
| FIGO stage |  |  |  |  |
| Ⅰ、Ⅱ | 5 | 1（3.03%） | 4（12.12%） | 0.0453 |
| Ⅲ、Ⅳ | 28 | 18（54.55%） | 10（30.30%） |  |
| Cisplatin response |  |  |  |  |
| Sensitive | 24 | 11（33.33%） | 13（39.39%） | 0.0163 |
| Resistant | 9 | 8（24.24%） | 1（3.03%） |  |

**Sup. Table 2. The primers used for quantitative RT-PCR**

| Gene | Sequence |
| --- | --- |
| CYLD | Forward: TTGGCAACTGGGATGGAAGA |
|  | Reverse: TCCTTTCCTGCGTCACACTC |
| EGFR | Forward: GACAGGCCACCTCGTCG |
|  | Reverse: CCGGCTCTCCCGATCAATAC |
| HER2 | Forward: CCGGAGCCGCAGTGAG |
|  | Reverse: TGTGCCGGTGCACACTTG |
| HER3 | Forward: CTCCGCTTGACTCAGCTCAC |
|  | Reverse: ATCTCAGCATCTCGGTCCCT |
| HER4 | Forward: GTTCAGGATGTGGACGTTGC |
|  | Reverse: CTGCCGTCACATTGTTCTGC |
| GAPDH | Forward: TGAACGGGAAGCTCACTGG |
|  | Reverse: TCCACCACCCTGTTGCTGTA |
| ABCB1-Site1 (ChIP) | Forward: CAGCTGATGCGCGTTTCTCTACTTG |
|  | Reverse: GCCTCACCACAGATGACTGCTCCCG |
| ABCB1-Site2 (ChIP) | Forward: CAGATTGCACGTACTTTTC |
|  | Reverse: TTTTCCAGGGCATTTT |

**Sup. Table 3. The antibodies used for Western blotting or Immunohistochemistry**

| Protein | Company | Catalog Number | Application |
| --- | --- | --- | --- |
| CYLD | Cell Signaling Technology | #8462s | WB, IHC |
| HER3 | Abways | CY5270 | WB |
| RIP | Abways | CY6582 | WB |
| Bcl-2 | Abways | CY5032 | WB |
| Bax | Abways | CY5059 | WB, IHC |
| Bcl-XL | Abways | CY5050 | WB, IHC |
| Survivin | Abways | CY5070 | WB |
| Bcl-3 | Proteintech | 23959-1-AP | WB |
| ABCB1 | Cell Signaling Technology | #13342s | WB |
| ABCB9 | ZEN Bioscience | 860017 | WB |
| ABCC1 | Cell Signaling Technology | #72202s | WB |
| ABCG2 | Abgent | AP1490c | WB |
| ABCB1 | Proteintech | 22336-I-AP | IHC |
| GAPDH | Bioword | AP0063 | WB |
| β-actin | Bioword | AP0060 | WB |
| p-STAT3(Thr705) | Cell Signaling Technology | #9145s | WB |
| STAT3 | Cell Signaling Technology | #9139s | WB |
| Ki-67 | Proteintech | 27309-I-AP | IHC |
| p-p38 | Cell Signaling Technology | #4511s | WB |
| p-Erk(p44/42MAPK) | Cell Signaling Technology | #4370s | WB |
| p-c-Jun(Ser73) | Cell Signaling Technology | #3270s | WB |
| p65 | Cell Signaling Technology | #8242 | WB |
| p-p65(ser536) | Cell Signaling Technology | #3030 | WB |

**Sup. Table 4. The primers used for plasmid construction**

| Plasmid | Sequence |
| --- | --- |
| sgHER3 | Forward: CACCGCACTGTACAAGCTCTACGAGG |
|  | Reverse: AAACCTCGTAGAGCTTGTACAGTGC |
| shCYLD#1 | Forward:CCGGGAAGAAGGTCGTGGTCAAGGTCTCGAGACCTTGACCACGACCTTTTCTTTTTG |
|  | Reverse:AATTCAAAAAGAAGAAGGTCGTGGTCAAGGTCTCGAGACCTTGACCACGACCTTCTTC |
| shCYLD#2 | Forward:CCGGGCCCAATACCAATGGAAGTATCTCGAGATACTTCCATTGGTATTGGGCTTTTTG |
|  | Reverse:AATTCAAAAAGCCCAATACCAATGGAAGTATCTCGAGATACTTCCATTGGTATTGGGC |
